# Hybridization between *Aedes aegypti* and *Aedes mascarensis* mosquitoes leads to disruption of male sex determination

**DOI:** 10.1038/s42003-024-06560-4

**Published:** 2024-07-22

**Authors:** Jiangtao Liang, Lin Kang, Pawel Michalak, Igor V. Sharakhov

**Affiliations:** 1 Department of Entomology, Virginia Polytechnic and State University, Blacksburg, VA USA; 2 Fralin Life Science Institute, Virginia Polytechnic and State University, Blacksburg, VA USA; 3 Center for Emerging, Zoonotic, and Arthropod-borne Pathogens, Virginia Polytechnic and State University, Blacksburg, VA USA; 4 Department of Biomedical Research, Edward Via College of Osteopathic Medicine, Monroe, LA USA; 5 College of Pharmacy, University of Louisiana Monroe, Monroe, LA USA; 6 Center for One Health Research, VA-MD College of Veterinary Medicine, Blacksburg, VA USA; 7 Institute of Evolution, University of Haifa, Haifa, Israel; 8 Department of Genetics and Cell Biology, Tomsk State University, Tomsk, Russia

**Keywords:** Evolutionary genetics, Genetic hybridization, Speciation

## Abstract

Understanding the sex determination pathway and its disruptions in mosquitoes is critical for the effective control of disease vectors through genetic manipulations based on sex separation. When male hybrids of *Aedes aegypti* females and *Ae. mascarensis* males are backcrossed to *Ae. aegypti* females, a portion of the backcross progeny manifests as males with abnormal sexual differentiation. We discovered a significant correlation between pupal abnormalities and the feminization of subsequent adults exemplified by the relative abundance of ovarian and testicular tissues. All intersex individuals were genetic males as they expressed a male determining factor, *Nix*. Further, our analysis of the sex-specific splicing of *doublesex* and *fruitless* transcripts demonstrated the presence of both male and female splice variants indicating that sex determination is disrupted. A comparative transcriptomic analysis revealed similar expression levels of most female-associated genes in reproductive organs and carcasses between intersexual males and normal females. Moreover, intersexes had largely normal gene expression in testes but significant gene downregulation in male accessory glands when compared with normal males. We conclude that evolving hybrid incompatibilities between *Ae. aegypti* and *Ae. mascarensis* involve disruption of sex determination and are accompanied by changes in gene expression associated with sexual differentiation.

## Abstract (web summary)

A study of intersexual mosquito males produced by hybridization between *Aedes aegypti* and *Ae. mascarensis* identified disruption of the sex determination pathway.

## Introduction

Speciation commences when the gene exchange between two populations is reduced and completes when multiple reproductive barriers accumulate. Taking into account the timing of occurrence—whether before or after zygote formation—and whether influenced by the environment or not, these barriers can be categorized as pre-zygotic and post-zygotic, as well as extrinsic and intrinsic isolations, respectively^1^. Among them, the intrinsic post-zygotic isolation gains more attention due to its relatively distinct phenotypes and its common occurrence^2^. The main manifestation of intrinsic postzygotic isolation is hybrid sterility or inviability, caused by epistatic interactions between multiple genes, consistent with the Bateson–Dobzhansky–Muller model^3–5^. Even though the complexity of gene misregulation in hybrids makes it difficult to identify genes that directly contribute to hybrid incompatibilities, as only a few such genes have been found thus far^6–8^, several broader patterns of reproductive isolation related to the interaction between sex chromosomes and autosomes have been described. First, it has been observed that hybrid sterility or inviability often occurs in the heterogametic sex (XY or ZW type), a phenomenon known as Haldane’s rule^9^. Second, the so-called large-X effect, arises when sterility or inviability of heterogametic hybrids is disproportionately caused by genes located on the X chromosome rather than autosomes^10,11^. Third, dubbed as Darwin’s corollary to Haldane’s rule, states that F1 hybrids from reciprocal crosses of different species typically exhibit varying degrees of sterility or lethality, possibly associated with interspecific differences in relative rates of evolution for autosomal vs. nonautosomal loci^12^.

Mosquitoes provide an attractive system for the study of speciation and hybrid incompatibilities. They belong to the Culicidae family that includes ~3500 species, many of which form species complexes with their members readily hybridizing despite a wide range of reproductive isolation forms. However, detailed developmental and evolutionary genetics studies of hybrid incompatibilities in mosquitoes are yet to be performed. There are two subfamilies within Culicidae: Anophelinae, which contains the genus *Anopheles* with species that can transfer human malaria, and Culicinae, which encompasses the genera *Aedes* and *Culex*, including vectors of various viral infections in humans^13^. Specifically, *Ae. aegypti* transmits arboviruses that cause Zika, dengue, yellow fever, and Chikungunya^14^. Exploring the mechanisms of hybrid incompatibilities and the associated genes in mosquitoes holds practical significance, offering potential applications in species-specific vector control.

Hybrid male sterility tends to evolve more rapidly than hybrid female sterility or hybrid inviability and is commonly observed in closely related species^15,16^, including mosquitoes^17^. Distinct phenotypes contributing to hybrid male sterility are typically associated with malfunction of male internal reproductive organs in *Anopheles* species^18,19^. Multiple studies of hybrids from crosses between species of the *An. gambiae* complex have shown various degrees of testicular atrophy and sperm abnormality^18–21^. Furthermore, hybridization can also affect somatic parts of the reproductive tract, such as male accessory glands (MAGs) and the ejaculatory duct^22^. However, male genitalia remain unaffected in F1 hybrids or backcrosses allowing for successful mating. At the cytological level, failures of premeiotic and meiotic processes can lead to the malformation of testis and sperm in *Anopheles* hybrid males^19^. Genetic mapping studies in *Anopheles* species demonstrated the large X-effect on male sterility and incompatible X-autosomal interactions in hybrid males^20,21,23^.

*Anopheles* mosquitoes have heteromorphic (X and Y) sex chromosomes, while *Aedes* mosquitoes have homomorphic sex-determining chromosomes that are morphologically indistinguishable between the sexes^24^. The determination of maleness in *Anopheles* and *Aedes* species is governed by distinct sex-determining factors. In *An. gambiae*, maleness is determined by the *Yob* gene (also known as *gYG2*) located on the Y chromosome^25,26^. In contrast, in *Ae. aegypti* and the Asian tiger mosquito *Ae. albopictus*, maleness is determined by the *Nix* gene, which is located within the M locus on the homomorphic chromosome 1^27,28^. Such cytogenetic diversity raises a question about the role played by sex chromosomes and sex determination systems in the variation of hybrid incompatibilities between these two genera. Notably, Haldane’s rule and Darwin’s corollary apply to both *Anopheles* and *Aedes*. However, Haldane’s rule applies to both sterility and inviability in *Anopheles*, but only to sterility in *Aedes*^17^, suggesting that dominance plays a role in evolution of hybrid inviability, while faster male evolution causes Haldane’s rule for sterility.

To understand the mechanisms of hybrid incompatibility and identify the underlying genetic factors, it is essential to accurately describe the phenotypes involved. Studies of hybrids between species of *Aedes* have identified incompatibility phenotypes that differ from those observed in *Anopheles* hybrids. Crosses between males of *Ae. hendersoni* or *Ae. brelandi* and females of *Ae. triseriatus* produced hybrid sterile males, described as intersexes, while female hybrids and males from the reciprocal crosses were morphologically normal and fertile^29,30^. Although intersex individuals had female spermatheca in their reproductive tracts, they still exhibited normal spermatogenesis, as demonstrated by the presence of mature sperm in the *vas deferens*. Abnormalities in the male genitalia, such as failure to complete rotation, the development of a second set of genitalia growing from abdominal sternite VIII, or the development of female cerci, caused sterility of adult intersexes^30^. It is worth noting that intersexes were observed in both male and female hybrids resulting from crosses between *Ae. brelandi* or *Ae. hendersoni* males and *Ae. zoosophus* females^31^. Hybrid breakdown was observed in backcross generations produced by *Ae. aegypti* females and F1 males resulting from crosses between *Ae. aegypti* females and *Ae. mascarensis* males, with progeny from all other backcross directions being normal^32^. A portion of backcross adult males exhibited abnormalities, including unrotated VIIIth and posterior abdominal segments, a malformed VIIIth sternite, and intersex phenotypes with intermediate external dimorphic characters such as genitalia, antenna, and maxillary palps, as well as with presence of spermathecae in the reproductive tract^32^. However, it is unclear whether feminization affects only adults or stages before the eclosion, such as pupa, since intersexes have not been studied at the earlier stages of the mosquito development. Furthermore, it is unknown whether feminization affects internal reproductive organs and other body parts in intersex mosquitoes. Therefore, the extent of phenotypic abnormalities, particularly in germline tissue, resulting from hybridization of *Ae. aegypti* and *Ae. mascarensis*, requires further study. To our knowledge, the feminization of hybrid males has not been observed as an outcome of interspecies hybridization among *Anopheles* mosquitoes. This suggests the presence of a different mechanism of reproductive isolation within *Aedes* species.

*Aedes aegypti* and *Ae. mascarensis* are closely related mosquito species of the Aegypti Group that originated in the southwestern Indian Ocean^33,34^. Multiple closely related species of the group have been described on the Indian Ocean islands and on the African continent, including *Ae. mascarensis* (Mauritius island), *Ae. pia* (Mayotte), *Ae. ‘aegypti’* Madagascar, *Ae. ‘aegypti’* Réunion, *Ae. ‘aegypti’* Mayotte, *Ae. ‘aegypti’* Europa, *Ae. aegypti formosus*, and *Ae. aegypti aegypti*^35^. The estimated divergence time of 8 million years ago between *Ae. aegypti* and *Ae. mascarensis* correlates with the Mauritius island formation. Post-speciation gene flow has also been detected between these two species^36^ and no fixed inversions have been found between them^37^. The genetic basis of intersexuality in hybrid *Aedes* males is not fully understood. One study suggested that incompatible interactions between the *Ae. mascarensis* male-determining chromosome 1 and homozygous *Ae. aegypti* genes caused intersexual development^32^. Using three morphological marker loci and five isozyme loci of the *Ae. aegypti* RED strain, a subsequent study linked chromosome 1 and chromosome 3 to external intersex phenotypes, while chromosome 2 was implicated in genitalia development^34^. Notably, the *Ae. mascarensis* chromosome 1 harbors *Nix*, a primary male determination gene that has been identified in multiple species of the genus *Aedes*^38^. It is possible that disruption of gene regulation in *Aedes* male backcrosses could be a mechanism of intersexual development, as gene expression regulation is based on interactions between loci. However, no studies have been conducted on gene expression in crosses between *Ae. mascarensis* and *Ae. aegypti*.

Previous research on hybrid incompatibility in *Aedes* mosquitoes has focused on the external phenotypes of adults^32,34^, thus, limiting understanding of developmental abnormalities associated with intersexuality. Here, we provide a rigorous survey of intersex traits, specifically hybrid incompatibility phenotypes resulting from crosses between *Ae. aegypti* and *Ae. mascarensis*. The study aims to characterize two distinct and stable hybrid incompatibility phenotypes: genital lobe shapes at the pupa stage and reproductive organ morphologies (both internal and external) at the adult stage. Adult hybrid males exhibited mixed sexual characteristics in the internal reproductive organs, including the presence of both testes and ovaries. We analyzed the sex-specific transcript splicing of key regulatory genes of the sex determination pathway and underlying patterns of genome-wide gene expression. Our findings provide the initial evidence of a strong association between hybrid incompatibility and male sex determination. Despite expressing the male-determining factor *Nix*, intersexual males showed disrupted splicing of *doublesex* and *fruitless* genes, leading to the production of both male and female variants. This research links the sex determination pathway, which usually consists of only a few genes, to reproductive isolation. This lays the foundation for identifying hybrid incompatibility genes, new sex determination genes, and mechanisms of reproductive isolation between species.

## Results

### Morphological abnormalities in backcross hybrid males at the pupal stage

**Fig. 1:**
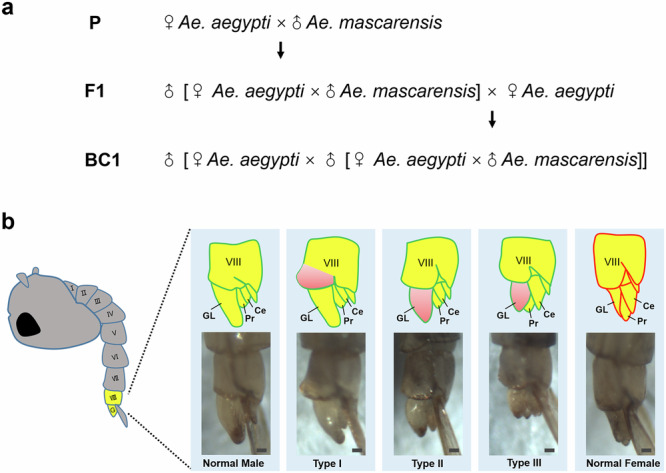
A crossing scheme and pupal morphology in the backcross generation. **a** A crossing scheme to obtain abnormal male individuals in a backcross generation 1 (BC1). **b** A morphological comparison of the VIIIth abdominal segment and genital parts between the normal males, abnormal individuals of Types I–III, and normal females at the pupa stage. All pictures were captured from a lateral view using a BC1 progeny from the cross using the *Ae. aegypti* Uganda strain. The filled yellow color highlights the position of the VIIIth abdominal segment and genital parts, while the filled pink colors indicate regions of abnormal morphology. I–VIII first to eighth abdominal segments, G genital parts, GL genital lobe, Pr Proctiger, Ce Cercus. Scale bars, 0.1 mm.

To investigate the developmental dynamics and extent of abnormalities associated with hybrid incompatibilities in *Aedes* mosquitoes, we obtained backcross progeny of generation 1 (BC1) by following the crossing scheme of the previous study^33^. Female *Ae. aegypti* (the Uganda or RED strain) were crossed with male *Ae. mascarensis* to produce F1 hybrid males. The F1 males were then backcrossed to female *Ae. aegypti* (Fig. 1a) to obtain either UUM (using Uganda) or RRM (using RED) BC1. We have confirmed the published results^33^ that a substantial number of abnormal males can only be obtained through this direction of backcrossing. Also, we occasionally observed a few abnormal males in the F1 generation from the *Ae. aegypti* × *Ae. mascarensis* cross, as was previously documented^32^, but these individuals were not included in this study. Based on our observations of mosquito development, abnormal phenotypes first appear at the pupa stage (Fig. 1b). In comparison to a normal male, which has long curved genital lobes, short cerci tightly attached to the proctiger, and a flat VIIIth abdominal segment, abnormal male pupae have malformations of either the VIIIth abdominal segment or genital parts. We classified abnormal male pupae into three types with respect to the level of abnormality. Type I is defined as a slightly abnormal phenotype, characterized by a bump on the ventral side of the VIIIth abdominal segment, but with normal genital lobes and cerci. Type II is a moderately abnormal phenotype, where the genital lobes are slightly shorter than usual, but still longer than the proctiger, while cerci appear normal. Type III is characterized as a highly abnormal phenotype, distinguished by extremely short genital lobes, which may be equal to or shorter than the proctiger; some individuals may also have elongated cerci. The shortening of the genital lobe in association with the elongation of the proctiger and cerci in Types II and III (Fig. 1b) indicates the increased feminization level of the abnormal male pupae. A normal female has the relatively longer proctiger and cerci than the genital lobe in comparison with a normal male. We recorded the numbers of female, normal male, and abnormal male pupae of each type in backcross generations across eight BC1 progenies (3 UUM and 5 RRM) (see ‘Methods’ and Supplementary Data 1) to control for potential differences in phenotype viability, using the aforementioned descriptions. The female to male sex ratio in BC1 using *Ae. mascarensis* and either *Ae. aegypti* Uganda or RED strains ranged from 0.95 to 1.19, indicating no obvious sex-biased lethality, and the proportion of morphological abnormalities varied from 7.56 to 19.51% among the eight backcross progenies.

### External and internal abnormalities in backcross hybrid males at the adult stage

We examined the abnormalities of both external and internal morphologies in adult males of backcrosses between *Aedes* mosquito species. No external abnormalities were observed in male adults that emerged from normal pupae. During the first 24 h after emergence of adult males, the VIIIth and posterior abdominal segments normally undergo a 180° rotation. As expected, this inversion was observed in all normal males. In comparison, adult males emerged from all abnormal pupae exhibited various abnormalities, such as a failure of 180° rotation of the VIIIth abdominal segment, malformation and feminization of reproductive organs. However, our crossing experiments revealed only minimal abnormalities in the morphology of antennae and palps, unlike the findings of a previous study^32^. To investigate the rotation of the VIIIth abdominal segment, we randomly selected 40 normal male pupae and compared them with 60 abnormal male pupae from one progeny of the UUM backcross (Supplementary Data 2). We found that all adult males who emerged from 40 normal pupae were able to rotate their VIIIth abdominal segment successfully after 24 h (Supplementary Fig. 1a). Consistent with previous studies^32^, no abdominal segment rotation was observed 24 h post-eclosion in all 60 abnormal males (Supplementary Fig. 1b). However, 13 out of 21 adults from Type I abnormal pupae were eventually able to rotate their VIIIth abdominal segments 48 h post-eclosion (Supplementary Data 2).

**Fig. 2:**
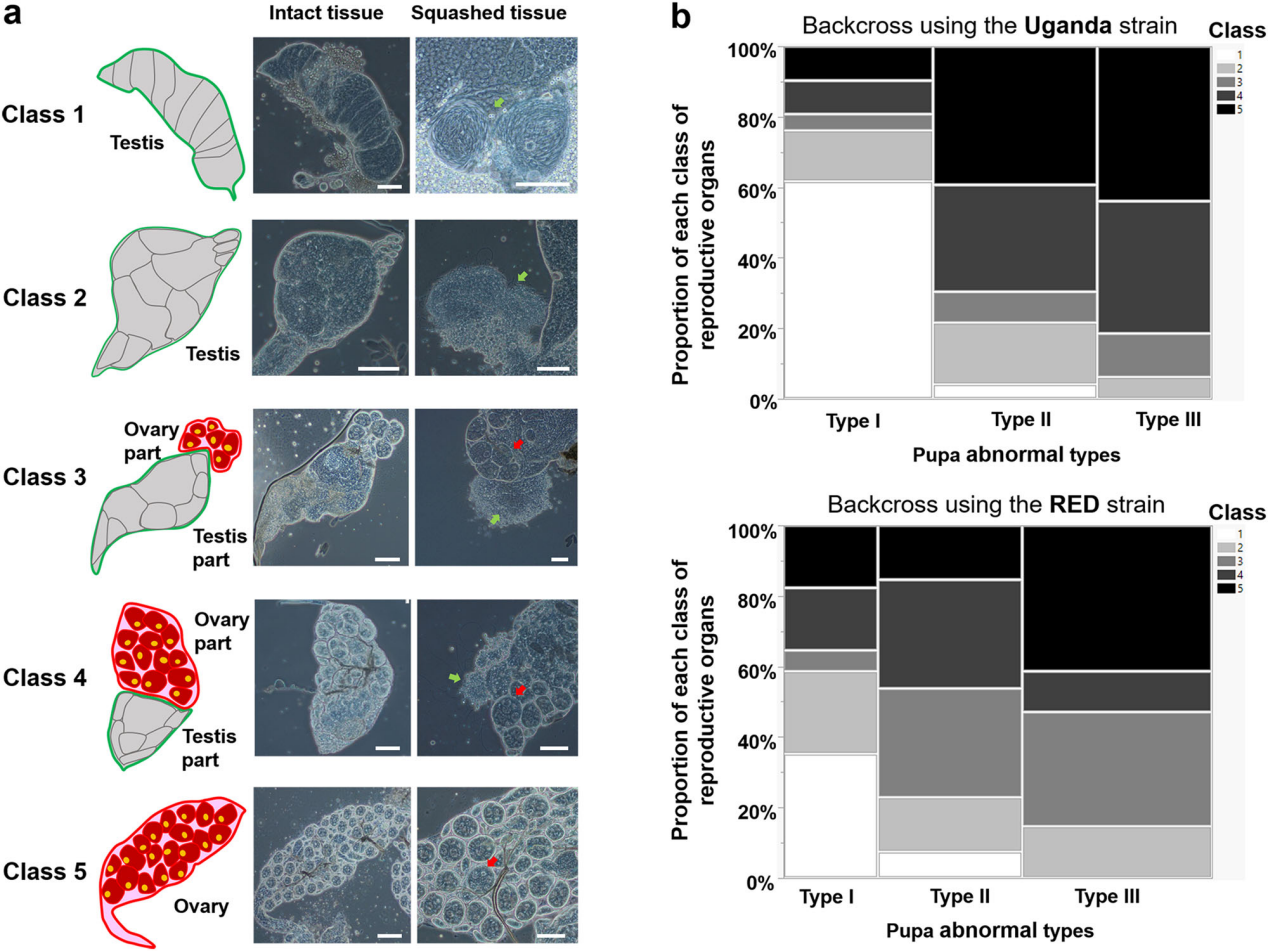
Classes of germline morphology in adults developing from abnormal male pupae. **a** Class 1 to Class 5 represent the abnormalities observed in the germline part of the reproductive organs. Green arrows indicate mature sperm, while red arrows indicate mature ovarioles. **b** Mosaic plots demonstrating the association between the abnormality levels at the adult and pupa stages in the backcrossing between *Ae. aegypti* Uganda (likelihood ratio test, *P* < 0.0001; Pearson test, *P* = 0.0002; *n* = 60) and *Ae. aegypti* RED (likelihood ratio test, *P* = 0.0012; Pearson test, *P* = 0.0016; *n* = 77). Scale bars, 100 µm.

After recording the external phenotypes, we dissected the same BC1 males to examine the morphology of their internal reproductive organs. No obvious abnormalities were found in reproductive organs of adults that emerged from normal pupae. However, we observed various abnormalities in both the germline and somatic parts of reproductive organs among individuals that emerged from abnormal male pupae. The germline morphology of 2–3-day old adult males emerged from abnormal pupae was classified into five classes (Fig. 2a). Class 1 had testes, identical to those of normal males, with clear layers of spermatocysts and motile sperm, visible after the testes were squashed. Class 2 had testes with no clear layers of spermatocysts but with motile sperm. Class 3 had reproductive organs consisting mostly of testis tissue and a minority of ovary tissue. Class 4 had reproductive organs consisting mostly of ovary tissue and a minority of testis tissue. Finally, Class 5 had only female reproductive organs, with ovaries present but testes absent. Therefore, the relative increase in the ovary component in Classes 3, 4, and 5 indicates an increased level of feminization in the abnormal adult males. Individuals from Classes 3, 4, and 5 were considered intersex due to the presence of female reproductive organs. Surprisingly, those with both ovary and testis tissues were able to produce mature ovarioles and sperm without any apparent impact on oogenesis and spermatogenesis (Supplementary Fig. 2 and Supplementary Movie 1). This suggests that the presence of both organs does not affect these processes. In the somatic parts of the reproductive system, intersex males were observed to have one to four spermathecae (Supplementary Fig. 3, Supplementary Data 2 and Supplementary Data 3), which is consistent with the findings of a previous study^32^. Female *Ae. aegypti* mosquitoes typically have three spermathecae: one median and two lateral. Normal males have a pair of MAGs in their reproductive system (Supplementary Fig. 3a). We observed intersex males with abnormally shaped and degenerated MAGs as well as the absence of one or both MAGs in (Supplementary Fig. 3, Supplementary Data 2 and Supplementary Data 3). Furthermore, we found instances of individuals with both spermathecae and MAGs (Supplementary Fig. 3c–f) or with different numbers of spermathecae (Supplementary Fig. 3g–i).

### Association between feminization levels of pupae and adults in backcross hybrid males

As shown above, the morphologies of abnormal male pupae and adults exhibited varying degrees of feminization. To test for an association between the feminization levels in pupae and adults, we recorded the number of abnormal pupae in each pupa type and the morphology, including MAG/spermatheca numbers and reproductive organ types, of adults that successfully emerged from each of these abnormal pupae. One backcross between *Ae. aegypti* Uganda and *Ae. mascarensis* (UUM) and five backcrosses between *Ae. aegypti* RED and *Ae. mascarensis* (RRM) were analyzed. There was a strong association between the feminization levels of backcross pupae and emerging adults in both UUM and RRM backcrosses.

In the progeny from UUM, 23.8% (5 out of 21) males from Type I, 78.26% (18 out of 23) from Type II, and 93.75% (15 out of 16) from Type III pupae developed ovaries in intersex males from Classes 3–5 (Supplementary Data 2). A significant association was observed between the proportion of males with different types of reproductive organs and the types of pupae they emerged from (likelihood ratio test, *P* < 0.0001 and Pearson test, *P* = 0.0002; Fig. 2b). This finding aligns with our observation that pupae with a stronger abnormality had an increased likelihood of giving rise to adults with more extensively feminized reproductive organs (greater proportion of the ovarian part). Similar results were obtained from the RRM progenies, with 41.18% (7 out of 17) males Type I, 76.92% (20 out of 26) Type II, and 85.29% (29 out of 34) Type III pupae (Supplementary Data 3), and a significant association between feminization levels of pupae and emerging adults (likelihood ratio test, *P* = 0.0012 and Pearson test, *P* = 0.0016; Fig. 2b).

The number of MAGs and spermathecae in the somatic parts of reproductive tracts in adult males were also significantly associated with the types of pupae they developed from in both UUM and RRM progenies (likelihood ratio and Pearson tests, *P* < 0.001; Supplementary Fig. 3j–m). Among abnormal UUM males, 76.19% (16 out of 21) of Type I pupae had two MAGs as in normal males, compared with 21.74% (5 out of 23) and 6.25% (1 out of 16) of those from Type II and III, respectively. The percentage of males without spermathecae, as in normal males, was 76.19% (16 out of 21) in Type I, 13.04% (3 out of 23) in Type II, and 6.25% (1 out of 16) Type III (Supplementary Fig. 3j, k and Supplementary Data 2). Similar trends were observed in the RRM offspring (Supplementary Fig. 3l, m and Supplementary Data 3).

### Expression of both male and female *doublesex* and *fruitless* splice variants in the abnormal backcross pupae and adults

**Fig. 3:**
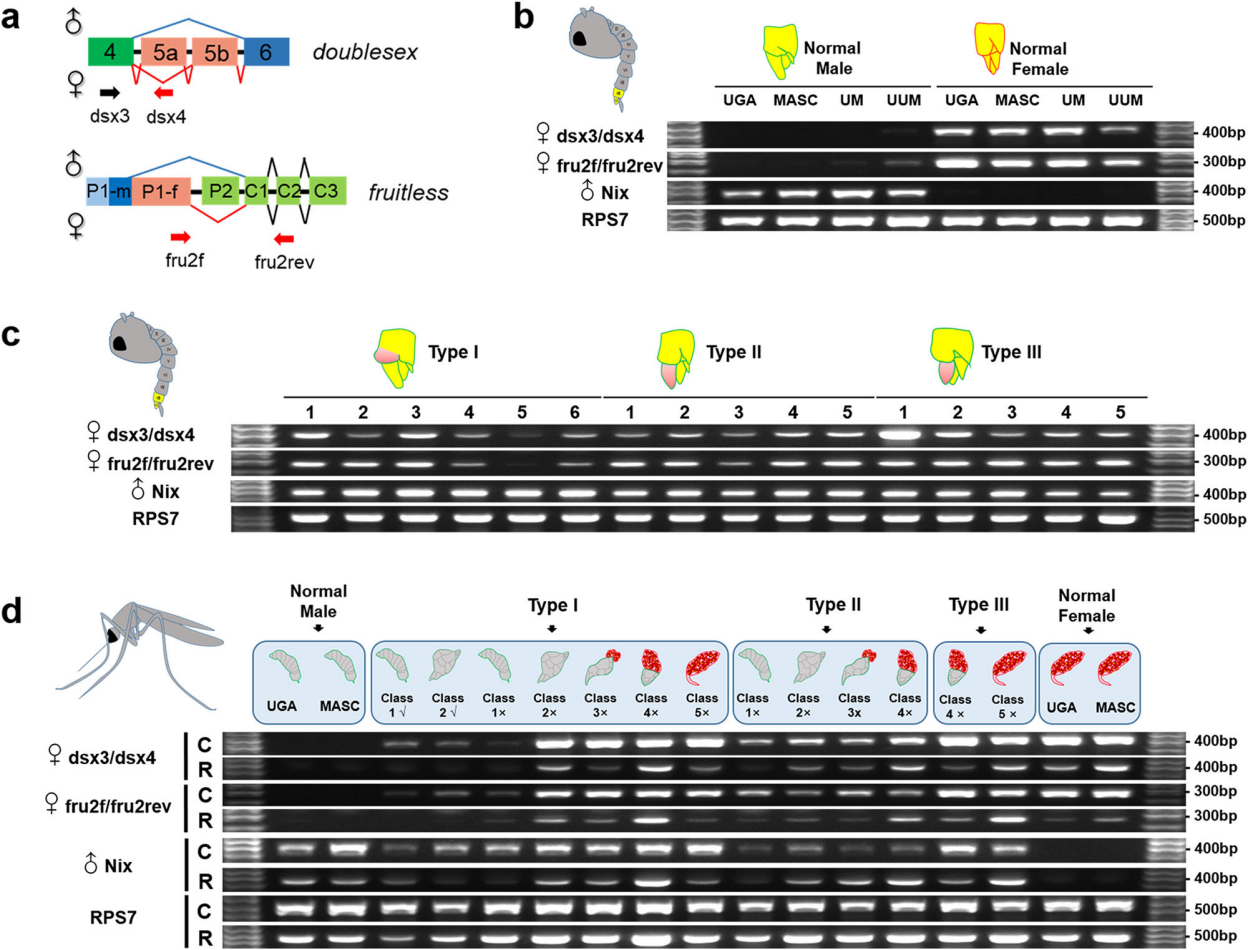
*Doublesex* and *fruitless* female-specific transcription in pupae and adults. **a** A scheme showing position of female-specific primers of *dsx* and *fru* genes. **b** RT-PCR results obtained on normal male and female pupae from pure species, F1 hybrids, and backcrosses. **c** RT-PCR results obtained on abnormal male pupae of Type I, Type II, and Type III. Numbers in each type indicate different individuals. **d** RT-PCR results obtained on abnormal adults emerging from different Types of pupae (I, II, III). *Nix* was used as a positive control for male-specific expression. *RPS7* was used as an endogenous control gene. UGA *Ae. aeypti* Uganda strain, MASC *Ae. mascarensis*, UM F1 hybrid between female UGA and male MASC, UUM backross generations from female UGA and male F1UM, √/×, successful/failed 180° rotation of VIIIth abdominal segment, C carcass, R reproductive organs.

Sex determination in most insects, including mosquitoes, is genetically determined through sex-specific splicing of the primary transcripts of *doublesex* (*dsx*) and *fruitless* (*fru*), two genes that encode highly conserved transcription factors, which program sexual dimorphism^39^. We hypothesized that the sex determination pathway in intersex males is misregulated with respect to sex-specific splicing of *dsx* and *fru*. Specifically, we expected to observe both male- and female-specific splice variants of the two genes in backcross hybrid males with strong feminization phenotypes. The *Nix* gene has been identified as a dominant male determination factor in *Aedes* mosquitoes. It is likely that the *Nix* gene interacts with other unidentified factors to regulate sex-specific splicing of *dsx* and *fru*^27,28,38^. The Nix proteins share a high degree of similarity, with a 97.6% identity over the entire 288 amino acid protein length, between *Ae. aegypti* and *Ae. mascarensis*^38^. Consequently, to verify the occurrence and timing of disruptions in the sex determination pathway, our focus was directed toward examining the expression of *Nix* and the splicing patterns of *dsx* and *fru* in abnormal males, juxtaposed with those in normal males and females (Fig. 3 and Supplementary Fig. 4).

To test if intersex males express *Nix*, we conducted reverse transcription polymerase chain reaction (RT-PCR) using previously published primers (Supplementary Data 4)^40^. We also performed RT-PCR using primers (Supplementary Data 4) for female and male transcripts of *dsx* and *fru* genes (Fig. 3a and Supplementary Fig. 4a) from the previous studies^41,42^. Female-specific transcripts of both *dsx* and *fru* were abundant in female pupae, but not in parental, hybrid F1 or normal backcross male pupae (Fig. 3b). A relatively weak gel band was produced by cDNA from normal backcross male pupae, indicating a very low level of female-specific transcription of *dsx* and *fru*. We then randomly selected 5–6 Type I–III pupae to examine transcription patterns of *dsx* and *fru*. As expected, we observed strong presence of female transcripts from both genes in all tested Type II and III. The presence of the female transcripts varied from strong to weak among individuals of Type I pupae (Fig. 3c). This outcome aligns with our phenotypic observations, indicating that higher-abnormality Types of pupae produce adults with more extensive feminization. Notably, *Nix* was strongly expressed in pupae individuals of all three Types (Fig. 3c).

To delve deeper into the expression patterns of *dsx* and *fru* in adults, we conducted RT-PCR using cDNA extracted from either the carcass or the reproductive organs of individual adult specimens. Similar expression patterns were observed in carcasses and reproductive organs of abnormal adults: strong bands of female-specific *dsx* and *fru* transcripts in adults from Classes 2-5 lacking the VIIIth abdominal segment rotation and emerged from Type I pupae, as well as all adults emerged from Type II and III pupae (Fig. 3d). As in pupae, *Nix* was expressed in all abnormal adults including those with ovarian tissue only in their reproductive tracts (Fig. 3d).

For comparison, using cDNA from the same samples as in Fig. 3, we tested the expression pattern of *fru* and *dsx* using primers spanning gene regions for both male and female splice variants (Supplementary Fig. 4a). We found that the *fru* and *dsx* transcripts show distinct sex-specific splicing patterns in pupae of pure species, F1 hybrids, and normal backcrosses (Supplementary Fig. 4b). Similar to what we found using female-specific primers, both male and female *dsx* and *fru* transcripts were abundant in Type II and III abnormal pupae implying feminization of males, while they were underrepresented in Type I pupae (Supplementary Fig. 4c). Although both male and female splice variants of *dsx* were present in carcasses and reproductive organs of most abnormal adult males, distinct female *fru* transcripts were absent in the carcasses of abnormal adults and reproductive organs of all individuals, including normal females (Supplementary Fig. 4d). This difference may have been due to stage- or tissue-specific expression patterns of *fru* in females. In contrast to the RT-PCR results obtained using cDNA from pupae, it is important to note that certain faint gel bands observed in the reproductive organs of adults may potentially be artifacts resulting from the lower quantity of RNA, particularly when extracted from limited tissue amounts. Nevertheless, the overall pattern of the sex-specific expression of *dsx* and *fru* transcripts demonstrated the presence of both male and female splice variants in the abnormal backcross pupae and adults confirming that they are intersexes.

### Groups of genes with distinct expression patterns in normal mosquitoes

We demonstrated that intersex individuals with both ovary and testis parts were able to produce mature ovarioles and sperm (Supplementary Fig. 2 and Supplementary Movie 1) and they also had 1–2 degenerated (or absent) MAGs along with the external abnormalities (Supplementary Fig. 1 and Supplementary Fig. 3). Therefore, we were interested in determining whether the gene expression patterns in their reproductive tracts and carcasses correlate with these abnormalities in sexual differentiation. Due to the diverse phenotypes involved in intersexual development, it is challenging to associate specific phenotypes with possible gene misregulation when considering all *Aedes* genes together. To address this challenge, we employed a more focused approach by comparing intersexual males with both normal BC1 and pure species individuals with respect to six distinct expression patterns: sex-specific/tissue-specific, sex-specific/tissue-biased, sex-specific/tissue-unbiased, sex-biased/tissue-specific, sex-biased/tissue-biased, and sex-biased/tissue-unbiased. To identify genes that display these six expression patterns, we developed a pipeline (Supplementary Fig. 5a), that used pair-wise comparisons of pure species transcripts (Supplementary Fig. 5b) to divide all genes into five expression clusters based on sex, species, and tissue. The obtained expression clusters are (1) Sex-specific/biased expression in reproductive organs (REPRODUCTIVE_SEX); (2) Species-specific/biased expression in reproductive organs (REPRODUCTIVE_SPECIES); (3) Sex-specific/biased expression in carcass (CARCASS_SEX); (4) Species-specific/biased expression in carcass (CARCASS_SPECIES); and (5) Tissue-specific/biased expression (TISSUE) (see ‘Methods’ and Supplementary Data 5). Using these gene clusters, we formed six groups of genes to examine potential gene misregulation in intersexual males. For instance, we identified 202 female-specific genes in the REPRODUCTIVE_SEX expression cluster and 900 reproductive-specific genes in the TISSUE expression cluster (Supplementary Data 6) to create a group of genes with female-specific/reproductive-specific expression in normal mosquitoes. We identified 83 genes shared between the two clusters representing the female-specific/reproductive-specific group. We used these transcriptomic data to analyze gene expression in intersexual males. These data can also provide valuable insight into *Ae. aegypti* and *Ae. mascarensis* biology and will serve as a foundation for future research projects.

### The gene expression patterns in intersexual males

For the analysis of transcription, we chose adult intersex males from classes 3 and 4 (see Fig. 2a). To better understand variation in our transcriptome dataset, we conducted a principal component analysis on RNA-seq Fragments Per Kilobase of transcript per Million mapped reads (FPKM) values from all the samples (Supplementary Fig. 6). The RNA-seq FPKM values of intersex individuals’ reproductive organs exhibit a distinct expression pattern, clustering separately between those of normal males and females. In comparison, FPKM values from the carcasses of pure species males and females, normal backcross males and females, and intersex individuals cluster together. To analyze the gene expression in intersexual males, we used the six groups of genes with diverse expression patterns in normal mosquitoes: sex-specific/tissue-specific, sex-specific/tissue-biased, sex-specific/tissue-unbiased, sex-biased/tissue-specific, sex-biased/tissue-biased, and sex-biased/tissue-unbiased (Supplementary Fig. 7). Genes from each of these group were used in five comparisons of expression in reproductive organs and carcasses of males, females and intersexes. These included two comparisons between normal individuals from pure species and the backcross (U vs. UUM and M vs. UUM) and three comparisons between normal and intersexual backcross individuals (UUM vs. UUMix, U vs. UUMix, and M vs. UUMix). We define the expression pattern of each gene as: ‘No change’ if it shows no differential expression in all five comparisons; ‘Downregulation’ or ‘Upregulation’ if it shows no differential expression in the first two comparisons but is significantly down- or upregulated in all three comparisons between normal and intersexual backcross individuals; and ‘Other’ if no consistent differential expression patterns exist in first two comparisons and/or last three comparisons. A summary and all the data for gene differential expression between intersexes and males or females are presented in Supplementary Fig. 8 and Supplementary Data 7–14. It is noteworthy that for all six groups of genes with female-specific or female-biased expression, the majority of genes showed the ‘No change’ pattern in reproductive organs of intersexual males when compared with reproductive organs of normal females (Supplementary Fig. 8a). Also, none of the six groups of genes exhibited an ‘Upregulation’ pattern in intersexes when compared to males or females regardless of the tissue (Supplementary Fig. 8). The potential reason is that, unlike normal males or females who express only one sex-specific transcripts, intersex individuals express both male and female transcripts, leading to relatively lower expression levels of sex-associated genes. In this study, we focused on the sex-specific/tissue-specific group of genes to profile expression patterns in adult intersexes. Expression heatmaps of sex-specific and carcass-specific genes in pure species and backcross individuals are shown in Supplementary Fig. 9.

### Expression of female-specific genes in intersexual males

**Fig. 4:**
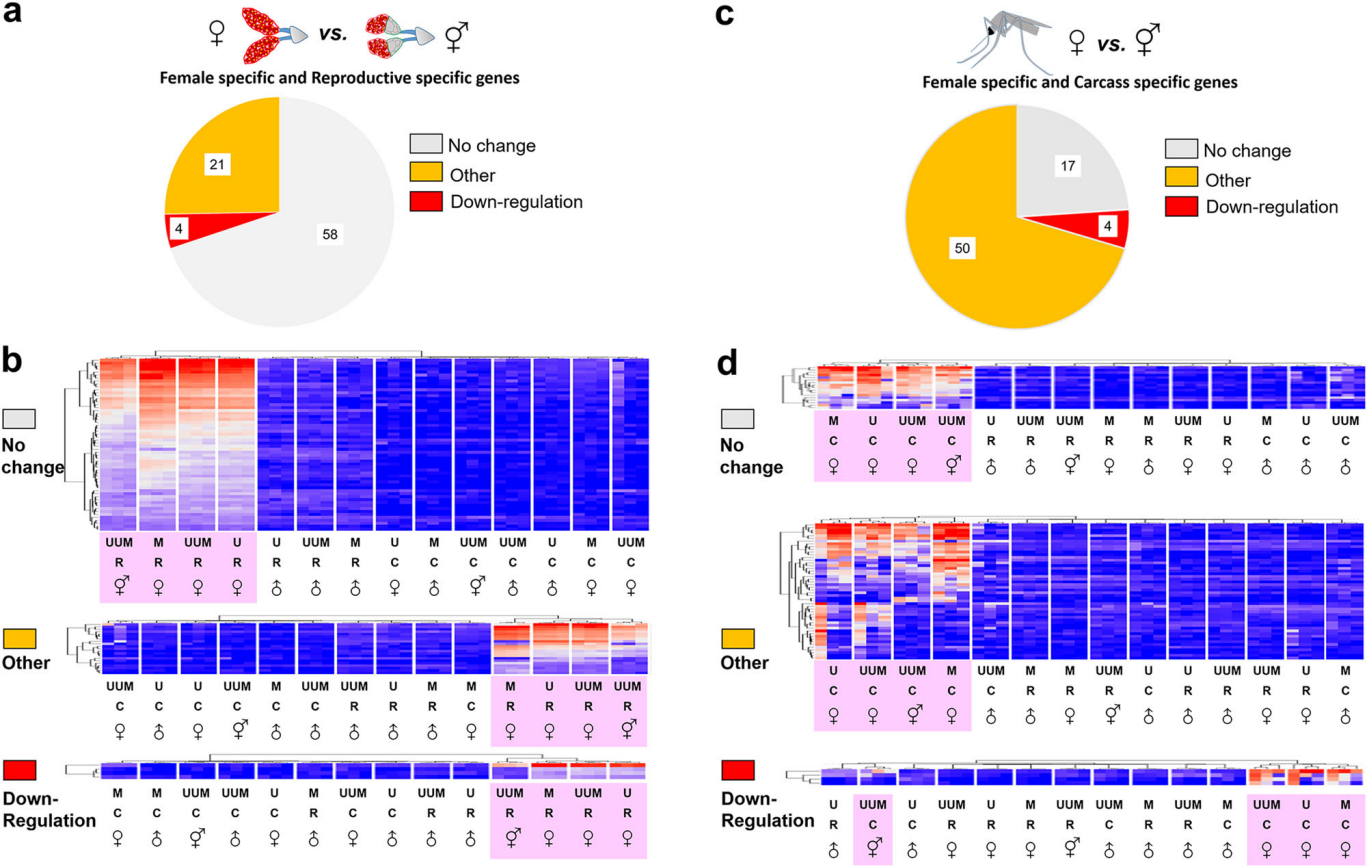
Expression patterns of female-specific and reproductive- or carcass-specific genes in intersexes. **a** A pie chart showing gene numbers with different expression patterns in the reproductive organ of intersexes compared with normal females. **b** Summary of gene expression heatmaps of genes in the reproductive organ of intersexes compared with normal females. **c** A pie chart showing gene numbers with different expression patterns in the carcass of intersexes compared with normal females. **d** Summary of gene expression heatmaps of genes in the carcass of intersexes compared with normal females. M *Ae. mascarensis*, U *Ae. aegypti* Uganda, UUM backcross generations, R reproductive organ, C carcass. The light pink background color indicates the samples associated with the expression group analyzed in this figure.

When comparing expression profiles in reproductive organs between intersexual males and normal females from pure species and backcrosses, 58 female-specific/reproductive-specific genes showed ‘No change’ in expression, 4 showed ‘Downregulation’, and 21 showed ‘Other’ expression patterns (Fig. 4a). Expression of many female-specific genes (Fig. 4b and Supplementary Fig. 9a–c) suggests a substantial feminization of reproductive organs in intersex individuals, which is consistent with our morphological findings. Many genes with the ‘No change’ pattern were previously described as ovary-specific or involved in oocyte development (Supplementary Fig. 5a and Supplementary Data 15 and  16a). Examples include AAEL000923 (unspecified product), AAEL007097 (4-nitrophenylphosphatase), and AAEL007584 (unspecified product)^43–45^. Our gene ontology (GO) term analysis further highlighted genes, AAEL004386 (chorion peroxidase), AAEL004388 (heme peroxidase), and AAEL004390 (heme peroxidase) as important for oogenesis (GO:0048477). Genes classified as ‘Other’ had expression patterns in intersexes that were more similar to females of both species and backcrosses than to corresponding males (Supplementary Fig. 9b and Supplementary Data 15 and 16b). For instance, AAEL000442, which encodes for maternal effect protein oskar and highly expresses in follicles^46^, and AAEL007657, a gene encoding a vitellogenin receptor and highly expressing in oocytes^47^, showed the ‘Other’ expression pattern. Also, AAEL008829 (unspecified product) and AAEL000961 (unspecified product), have been implicated in eggshell development^48^. Four genes with the ‘Downregulation’ pattern include AAEL003012 (protease m1 zinc metalloprotease), AAEL004199 (allergen, putative), AAEL005350 (unspecified product), and AAEL010055 (unspecified product) (Supplementary Fig. 9c and Supplementary Data 16c). Despite the limited functional annotation available for many female-specific/reproductive-specific genes, these results indicate a strong feminization of the germline in intersexual males.

Comparing female-specific/carcass-specific genes between intersexes and normal females, we found 17, 4, and 50 genes exhibiting ‘No change’, ‘Downregulation’, and ‘Other’ expression patterns, respectively (Fig. 4c). We observed abundant expression of female-associated genes in the intersex carcass (Fig. 4d and Supplementary Fig. 9d–f). Interestingly, most of these genes encode for female-enriched salivary gland proteins^49,50^. Out of the 17 genes with ‘No change’ expression pattern, 11 were salivary gland-associated genes according to the VectorBase annotation or published data^49,50^ (Supplementary Fig. 9d and Supplementary Data 15 and 16d). The three most highly expressed genes in this group, AAEL000533 (lectin-like protein), AAEL000749 (angiopoietin-like protein), and AAEL003182 (salivary serpin), are also immune system-related. In addition, two genes with ‘No change’ expression, AAEL010196 and AAEL013712, encode for trypsins that are associated with bloodmeal digestion in the female mosquito midgut^51,52^. Of the only four genes exhibiting the ‘Downregulation’ pattern, three genes displayed very low expression across the three replicates (Supplementary Fig. 9f and Supplementary Data 16f). They were AAEL001690 (serine-type endopeptidase), AAEL013118 (unspecified product), and AAEL009165 (unspecified product). Only one gene, AAEL000793 (venom allergen), which is associated with hematophagy^50^, had slightly elevated (but still lower than in normal females) expression in one of three replicates of the intersex carcass. Fourteen out of the top 20 genes with the ‘Other’ expression pattern are related to female-specific salivary gland proteins or midgut proteins (Supplementary Fig. 9e and Supplementary Data 15 and 16e). Another female-enriched transcript, AAEL005656, important for female flight^53^, was also expressed in intersexes. This gene is mainly expressed at the pupa stage^54^, but its expression was also high in adult carcasses of *Ae. aegypti* Uganda and backcross females, and much lower in *Ae. mascarensis* females.

### Misregulation of male-specific genes in intersexual males

**Fig. 5:**
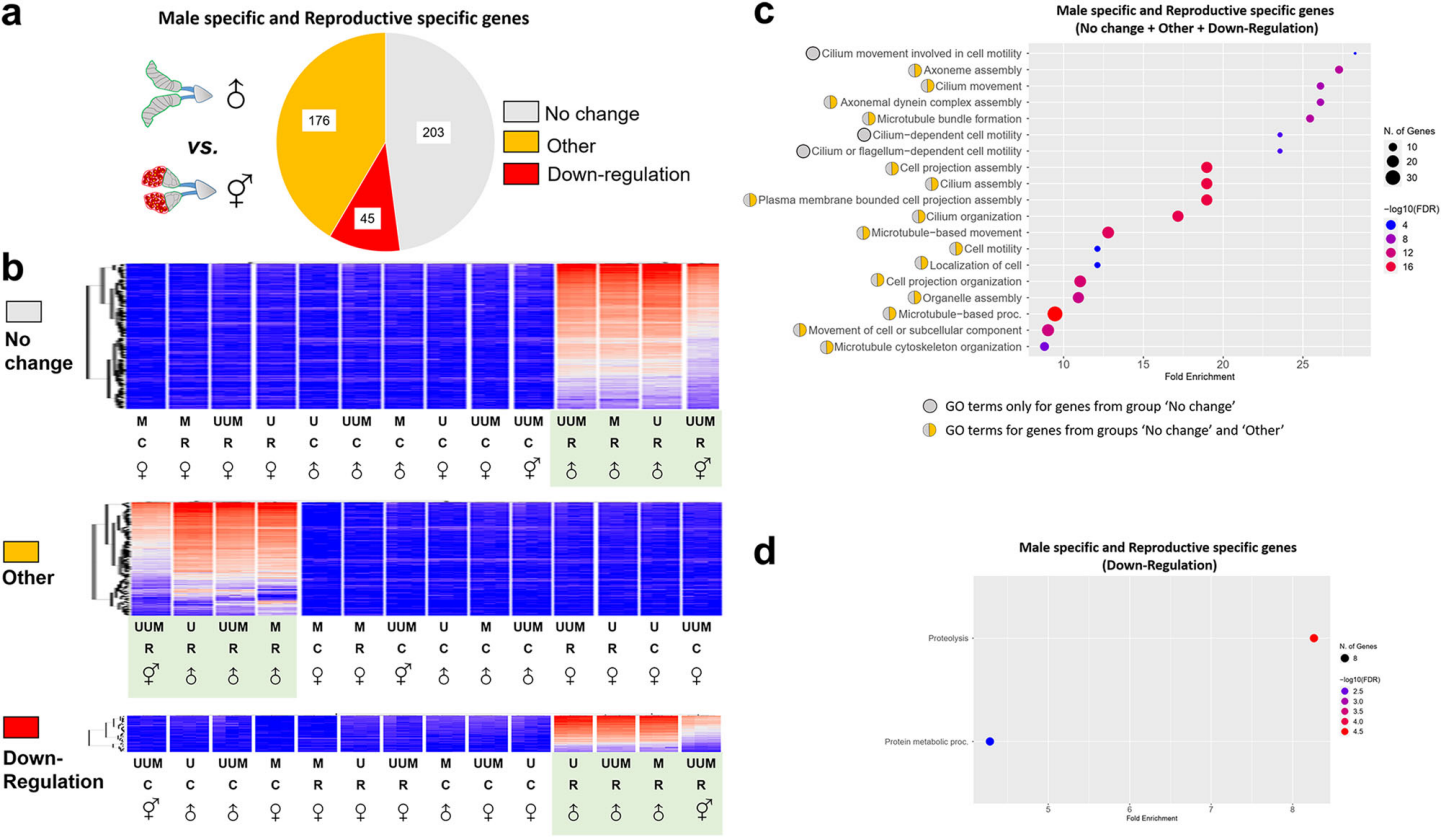
Expression patterns of male-specific/reproductive-specific genes in intersexes. **a** A pie chart depicting gene numbers with expression patterns in the reproductive organs of intersexes compared with normal males. **b** Summary of heatmaps for three types of expression patterns. **c** Enrichments of GO terms using all male-specific/reproductive-specific genes. **d** Enrichments of GO terms in ‘Downregulation’ pattern when all male-specific/reproductive-specific genes are used as a background. M *Ae. mascarensis*, U *Ae. aegypti* Uganda, UUM backcross generations, R reproductive organ, C carcass. The light green background color indicates the samples associated with the expression group analyzed in this figure.

Male internal reproductive organs of mosquitoes have two largest parts, MAGs and testes, that contribute most to the transcript abundance. Comparing male-specific/reproductive-specific genes between intersexes and normal males, we found 203, 176, and 45 genes exhibiting ‘No change’, ‘Other’, and ‘Downregulation’ expression patterns, respectively (Fig. 5a, b, Supplementary Fig. 9g–i, and Supplementary Data 16g–i and Supplementary Data 17). As expected, our GO term enrichment analysis of all male-specific/reproductive-specific genes determined that they mainly have functions in testes associated with sperm motility, such as microtubule bundle formation and cilium movement (Fig. 5c and Supplementary Data 17a). In addition, all enriched GO terms only include genes with the ‘No change’ pattern or both ‘No change’ and ‘Other’ expression patterns. Interestingly, genes important for cilium movement of sperm (GO:0060294, GO:0060285, and GO:0001539) show only ‘No change’ pattern, which is consistent with our observation of sperm motility in reproductive organs of intersexes (Supplementary Movie 1, Supplementary Fig. 2). Among 45 genes with the ‘Downregulation’ pattern in intersexes, 36 genes have been characterized as MAG-specific according to previously published RNA-seq data (Supplementary Data 18)^44^. Our GO term enrichment analysis using all male-specific/reproductive-specific genes as a background identified eight trypsin-associated genes, potentially taking part in proteolysis (GO:0006508) and protein metabolic process (GO:0019538) (Fig. 5d and Supplementary Data 17b). These results are consistent with our observation of missing or malformed MAGs in intersex reproductive organs (Supplementary Fig. 3).

In male carcasses, only six genes were found to be male-specific/carcass-specific (Supplementary Fig. 9j, k and Supplementary Data 16j–l). Two genes, AAEL008754 (unspecified product) and AAEL010201 (axonemal dynein intermediate chain), had ‘No change’ expression pattern when intersexes we compared with normal males. Four genes, AAEL021838 (myosin heavy chain), AAEL017539 (cytochrome P450), AAEL009682 (serine collagenase 1 precursor), and AAEL003079 (glucuronosyl transferase), had ‘Other’ expression pattern. AAEL021838 has been mapped to the M-locus and named *myo-sex* gene for its essential role in male flightiness^40,55^. *Myo-sex* had similarly higher levels of expression in intersexes and *Ae. mascarensis* males, but lower expression levels in *Ae. aegypti* males and normal backcross males (Supplementary Fig. 9k), in agreement with our backcrossing scheme, in which intersexes inherit the M-locus from *Ae. mascarensis*. No male-specific/carcass-specific genes had ‘Downregulation’ or ‘Upregulation’ patterns.

## Discussion

In this study, we tested if hybrid incompatibility in the *Ae. aegypti* / *Ae. mascarensis* backcross involves the formation of intersexes via disruption of the sex determination pathway. We studied intersexual males at the two levels: (1) sex determination by analyzing expression of *Nix* and sex-specific splicing of *dsx* and *fru*, and (2) sexual differentiation by studying morphological phenotypes in pupae and adults and genome-wide sex- and tissue-specific gene expression. Intersex is defined as an individual possessing a uniform genotype but exhibiting either a uniform or a mosaic phenotype^56^. Intersexuality is associated with malfunction of sex determination and sexual differentiation, which can be caused by chromosomal aberrations (e.g. polyploidy), loss-of-function mutations, epigenetic and environmental factors, as well as intra- and interspecies crosses^56,57^.

A rigorous phenotype description is a key step in characterizing hybrid incompatibilities and mapping the corresponding genetic factors. Previous genetic analyses involving crosses between *Ae. aegypti* and *Ae. mascarensis* have provided some support for the hypothesis that homozygous regions in the *Ae. aegypti* genome, located on both autosomes and sex chromosomes, interact with the male-determining M-locus of *Ae. mascarensis* to induce abnormalities in sexual differentiation^32,34^. However, the intersex phenotypes examined in these studies were limited to external malformations, such as antenna length and rotation of the VIIIth abdominal segment, and only one internal phenotype, the presence of spermatheca. Insufficient descriptions of phenotypes and a limited understanding of the sex determination pathway in *Aedes* mosquitoes have led to the inference that the sex-determining mechanism operates normally, but the control of differentiation of male genitalia is impaired^34^. Nevertheless, our study indicates that this conclusion is not supported by the evidence. In contrast to prior research, our work provides a more detailed description of the abnormalities observed in backcross males between *Ae. aegypti* and *Ae. mascarensis*. We documented hybrid incompatibilities evident at both pupal and adult stages, primarily characterized by the feminization of both external and internal reproductive tissues in abnormal males, referred to as intersexes. Utilizing these phenotypes, we hypothesized and substantiated the correlation between the disruption of the male sex determination pathway and the breakdown of male hybrids. This study presents direct evidence of the role of genetic factors evolved in male sex determination and sexual differentiation contributing to reproductive isolation in closely related species featuring homomorphic sex chromosomes when males are the heterogametic sex.

Instances of intersexes have been frequently reported in mosquitoes, particularly in various *Aedes* species, for diverse reasons. For example, thermal pressures can induce different levels of feminization in males of *Ae. stimulans*^58^*, Ae. sierrensis*^59^, *Ae. communis*^60^*, Ae. aegypti*^61^, and multiple other *Aedes* species^60^. Apart from the hybridization observed between *Ae. aegypti* and *Ae. mascarensis* as explored in our study, intersexual individuals can result from crosses between various species, subspecies, or strains within both *Aedes* and *Culex* genera. For example, feminized males have been reported in crosses between female *Ae. triseriatus* and male *Ae. hendersoni* or *Ae. brelandi*^30^. Reciprocal crosses between *Ae. triseriatus* and *Ae. zoosophus* also produce feminized males^31^. Moreover, both male and female intersexes can be found among *Ae. brelandi*/*Ae. zoosophus* and *Ae. hendersoni*/*Ae. zoosophus* hybrids. Female intersexes can be found in hybrids between *Cx. pipiens pipiens* strain 10 and *Cx. p. quinquefasciatus* strain Hanford^62^. Intersexuality caused by interspecies hybridization also occurs in the fruit flies *Drosophila repletae* and *D. neorepleta*^63^. In addition to mosquitoes and fruit flies, which both have an XY sex-determining system, hybrid intersexes (masculinization of genetic females) have been reported in crosses between different geographical strains of *Lymantria dispar*^64,65^ and between closely related species *Smerinthus ocellata* and *S. populi*^66^, moths with a ZW sex-determining system. Although the phenotypes and underlying mechanisms are not well understood, the prevalent occurrence of hybrid intersexes suggests potential roles of sex determination in reinforcement of the early stages of speciation in insects.

In most insects, sex is determined genetically and hierarchically with a fast-evolving master regulator gene, such as *Sex-lethal* in *Dr. melanogaster*, at the top of the cascade and the highly conserved *dsx* and *fru* genes at the bottom of the cascade^67,68^. In mosquitoes, two unrelated master genes contributing to male determination have been found, namely *Nix* in *Ae. aegypti*^27^ and *Yob* in *An. gambiae*^25^. The expression of *Nix* in all abnormal individuals in this study confirms their male genetic identity and initiation of the male determination pathway. The presence of both female and male transcripts of *dsx* and *fru* indicates that the normal sex determination pathway is disrupted in intersexual males. Furthermore, our finding suggests that genetic elements regulating sex-specific splicing of these two genes contribute to the abnormalities in sexual differentiation of male hybrids between *Ae. aegypti* and *Ae. mascarensis*. Our analyses of the morphology and gene expression of intersexes, as well as normal males and females from pure species and backcrosses, align with this hypothesis. Intersexual males had many female-specific transcripts in both germline tissues (e.g. ovaries) and somatic tissues (e.g. salivary glands and midgut) with female-like expression patterns. Another noteworthy observation is that there was no discernible alteration in the expression of genes associated with sperm movement. This suggests that spermatogenesis in intersexual males can progress normally, resulting in the production of mature sperm, consistent with our observation of actively motile sperm in the testis coexisting with mature ovarioles. At the same time, MAGs in intersex reproductive organs are often missing or malformed, which aligns with downregulation of MAG-specific genes in intersexual males. Mosquito MAGs produce seminal fluid proteins that are transferred to females during mating and play a large role in inducing post-mating responses including induction of gene expression in females^69,70^. Moreover, mRNA can be transferred with the seminal fluid during mating and translated into proteins in *Drosophila* females^71,72^. Our results suggest that important functions of MAGs is impaired in intersexual males.

**Fig. 6:**
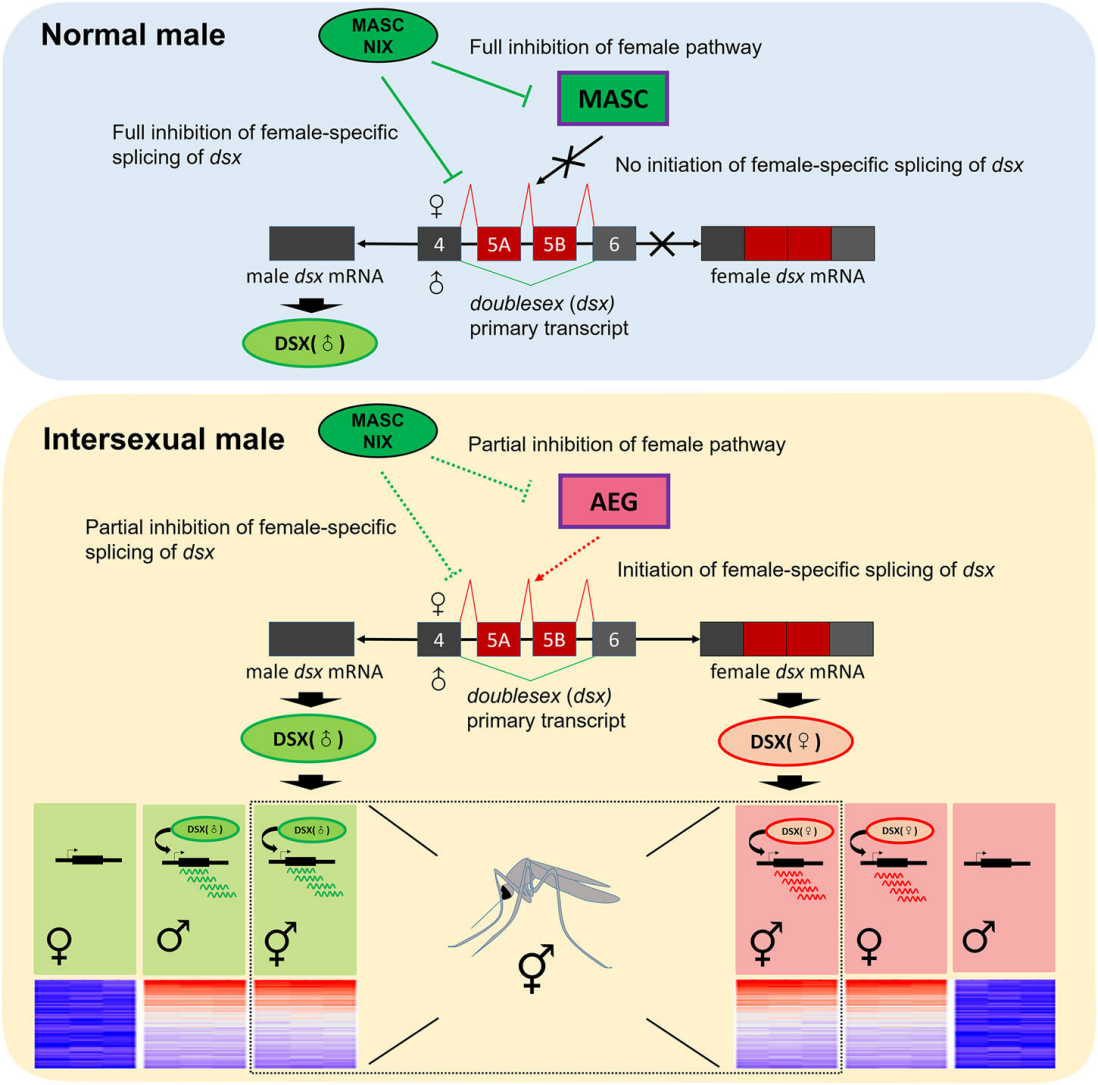
A hypothetical mechanism of the sex determination pathway misregulation in the intersexual male using *doublesex*, one of the two key transcription factors, as an example. In the normal male, interactions between the primary male determination factor the *Ae. mascarensis* Nix protein (MASC NIX) and its full inhibition of targets from the same species, intermediate step protein MASC (green color rectangle) and/or splicing sites of *doublesex* (*dsx*) primary transcript are shown. Only male *dsx* mRNA and protein (DSX) are produced to program male differentiation. In the intersexual male, abnormal interactions between the primary male determination factor the *Ae. mascarensis* Nix protein (MASC NIX) and its partial inhibition of targets from *Ae. aegypti*, intermediate step protein AEG (pink color rectangle) and/or splicing sites of *doublesex* (*dsx*) primary transcript are shown. The partial inhibition of female pathway protein(s) and splicing sites leads to production of female *dsx* mRNA and protein, which program female differentiation in a male genetic background. Below is shown the comparison of transcription heatmaps between intersexual males and normal males and females.

As genetic networks causing hybrid incompatibility typically involve complexity with multiple molecular pathways^7^, identifying the genes responsible for abnormalities requires performing a high-resolution mapping. Unlike *Anopheles* mosquito crosses, where the breakdown of hybrid males may be associated with the disruption of spermatogenesis in the testis^19^, we can narrow down the range of key hybrid incompatibility genes between *Ae. aegypti* and *Ae. mascarensis* to the sex determination pathway. Further support of the role of the sex determination pathway in formation of intersexes comes from the study of loss-of-function mutations in the master regulator. The knockout of *Nix* in *Ae. albopictus* can result in intersex males with ovaries^28^. In comparison, silencing of *Yob* in *An. gambiae* leads to male lethality rather than male feminization^25^. Additionally, it has been demonstrated that replacing the *An. arabienesis* Y chromosome, which includes the *Yob* gene, with that of *An. gambiae* in the *An. arabiensis* genomic background does not cause any apparent male insufficiency^73^. This difference may be explained by the tight association between the sex determination and dosage compensation pathways in *Anopheles* mosquitoes^74–76^. The regulators of sex-specific alternative splicing of *dsx* and *fru* emerge as strong candidates for the breakdown in the *Ae. aegypti*/*Ae. mascarensis* backcrosses (Fig. 6). In essence, the fast evolution of yet to be discovered genes in the sex determination pathway may be the main reason for hybrid incompatibilities in *Aedes* mosquitoes. Building upon this work, it will be possible to uncover new roles for previously identified regulators and unravel undiscovered regulators in the future. This endeavor is crucial for enhancing our comprehension of sex determination, sexual differentiation, and reproductive isolation in *Aedes*. Furthermore, given that intersexes have been observed in hybrids of closely related species across various insect groups, future investigations may shed light on whether sex determination is a necessary element in the reinforcement of speciation. It remains to be explored if sex determination is the primary or sole mechanism contributing to the early stages of reproductive isolation in closely related species, exemplified by *Aedes* and *Culex* mosquitoes and moths.

Intersexes can serve as a valuable model for studying genetic factors involved in sex determination, sexual differentiation, mating, host-seeking, and blood-biting behaviors in mosquitoes. Research on hybrid malfunctions in mosquitoes can help identify potential genes relevant to vector control. For example, a recent study demonstrated that targeting sex determination in *Ae. aegypti* is a promising approach to suppress mosquito populations^77^. Finding new the sex determination pathway genes and their disruptions in mosquitoes will contribute to the effective control of disease vectors through genetic manipulations based on sex separation. Also, targeting female-specific genes identified in this study could be used to generate sterile females or non-blood-digesting females.

## Methods

### Mosquito species and strains

Two laboratory strains of *Aedes aegypti*, RED and Uganda, and a colony of *Ae. mascarensis* were used in this study. The RED strain carrying a red-eye (*re*) mutation was obtained from the D. Severson laboratory at the University of Notre Dame via the Z. Tu laboratory at Virginia Tech. The *Ae. aegypti* RED strain had been used in the studies by the groups of G.B. Craig and K.S. Rai to describe hybrid breakdown in the *Aedes* species^32,34^. The Uganda strain was obtained from A. Powers at the CDC and Z. Adelman from Texas A&M University via Z. Tu laboratory. The *Ae. mascarensis* colony was obtained from the J.R. Powell laboratory at Yale University. Originally, this *Ae. mascarensis* strain was collected by A. Bheecarry on Mauritius in December 2014; its colony has been established by J.R. Powell^35^. Mosquitoes were reared at 27 ± 1 °C, with a 12-h photoperiod and 70 ± 5% relative humidity. Larvae were fed with fish food. Adult mosquitoes were fed with 10% sugar water. To stimulate egg development, females were fed on defibrinated sheep blood using artificial blood-feeders.

### Backcrossing experiments

In order to obtain interspecies F1 hybrids, we selected each sex at the pupa stage according to the morphology of genital lobes^78^ and placed multiple males and females of different species in one cage for mass mating. After eclosion, they were allowed to mate for 2–5 days. The same approach was used for backcrossing experiments. To obtain abnormal backcross males, we followed the crossing scheme of the previous studies^32,34^. Accordingly, F1 males were obtained from the cross between female *Ae. aegypti* and male *Ae. mascarensis*. These F1 males were backcrossed to *Ae. aegypti* females and the progeny of this backcross were analyzed in this study. To obtain backcrosses using *Ae. aegypti* Uganda and *Ae. mascarensis* (the UUM backcross), we placed 60 Uganda females and 10 F1 males in one cage and gave mated females three blood-feedings to produce three UUM progenies (Supplementary Data 1). To obtain backcrosses using *Ae. aegypti* RED and *Ae. mascarensis* (the RRM backcross), we placed 20 RED females and 3 F1 males in five cages. Five RRM progenies (one from each cage) of the females that were blood-fed one time were obtained and recorded (Supplementary Data 1). Variations in numbers of mosquitoes taken for the backcross experiments (UUM vs. RRM) were due to differences in the availability of F1 males.

### Mosquito dissection and morphology observation

The assessment of mosquito external morphology and dissections were carried out using an Olympus SZ61 stereo microscope (Olympus, Tokyo, Japan). Males and females at the pupa stage were separated and adults after eclosion were collected, dissected, and photographed with an Olympus Q-Color5™ imaging system (Olympus, Tokyo, Japan). Internal reproductive organs were dissected, placed on a slide in 1× PBS, and covered with a coverslip. Any extra tissue and solution on the slide were carefully removed with filter paper. We observed the morphology of internal reproductive organs using an X41 phase contrast microscope (Olympus, Tokyo, Japan), and recorded images and movies with a UC90 digital camera (Olympus, Tokyo, Japan). The types of reproductive organs and the numbers of MAGs and spermatheca in abnormal males were recorded.

### Tissue dissection and total RNA extraction

To extract total RNA for sequencing, we dissected 1–3-day-old virgin adult mosquitoes. For each sample, 20 reproductive organs including germline and somatic parts were dissected out in 1× PBS and pooled together in one tube with a TRIzol™ reagent. In addition, for each pool of reproductive organs a corresponding single body carcass (without reproductive organs) was preserved in an individual tube with TRIzol™ reagent. Total RNA was extracted using a Direct-Zol^TM^ RNA MiniPrep Kit (Zymo Research, Irvine, California, USA), following the protocol provided by the company. Three biological replicates were prepared for both reproductive organs and carcasses dissected from females and males of pure species, *Ae. aegypti* Uganda and *Ae. mascarensis*, as well as normal females, normal males, and intersex males from the backcross progeny. Intersex males are chosen using abnormal category of classes 3 and 4 (see Fig. 2a). To extract total RNA for RT-PCR, individual pupae and the reproductive organ and the carcass of individual adult were processed with a Direct-ZolTM RNA MiniPrep Kit (Zymo Research, Irvine, California, USA).

### Reverse transcription polymerase chain reaction (RT-PCR)

Splice variant-specific gene expression was tested using a two-step RT-PCR. The cDNA was generated using a SuperScript™ IV First-Strand Synthesis System (Invitrogen, Carlsbad, CA, USA). Following the protocol provided by the company, 2 µl of RNA from the whole pupa body or adult carcass and 8 µl of RNA from individual adult reproductive organs were treated with ezDNase™ Enzyme to remove genomic DNA and then used for the reverse transcription reaction to produce first-strand cDNA. RNA has been removed with *E. coli* RNAseH, provided in the kit, before PCR amplification. Each 20 µl PCR mix consisted of 1 µl cDNA, 10 µl 2X Platinum^TM^ II Hot-start PCR Master Mix (Invitrogen, Carlsbad, CA, US), 0.5 µl of 10 µM forward and reverse primers, and 8 µl of water.

Several genes involved in the *Ae. aegypti* sex determination pathway were tested in this study, including *doublesex* (*dsx*) (VectorBase ID: AAEL009114), *fruitless* (*fru*) (VectorBase ID: AAEL024283), and *Nix* (VectorBase ID: AAEL022912). The 40 S ribosomal protein S7 (RpS7) gene (VectorBase ID: AAEL009496) was used as a loading control (primer information can be found in Supplementary Data 4). PCR was performed in the C1000 TouchTM thermal cycler platform (Bio-Rad Laboratories, Hercules, CA, USA) starting with a 2-min incubation at 94 °C followed by 34 cycles of 94 °C for 15 s, 61 °C for 15 s, 68 °C for 8 s (for primer pairs of dsx3/dsx4, fru2f/fru2rev, Nix and AaeRPS7) or 30 s (for primer pairs of dsx3/5 and fru1/fru3), 68 °C for 1 min, and a final hold at 4 °C. Afterwards, 6 µl of PCR products was mixed with 4 µl of 6× gel loading dye. Each 10 µl mixture was then run on a 1% agarose gel and registered with a Fotodyne Imaging Hood Ethidium Bromide Filter 60-2030 Camera (Fotodyne Inc., Hartland, WI, USA) using the same parameters for each gene.

### RNA sequencing

RNA-seq libraries were prepared from ~1.8 µg of RNA isolated from each individual mosquito carcass and from ~1.7 µg of pooled RNA isolated from 20 pairs of individual reproductive organs. Libraries of a total of 42 samples from 14 groups with three biological replicates per group were prepared and sequenced (Supplementary Data 19) using service at GeneWiz (South Plainfield, NJ, USA). Briefly, total RNA was first quantified and assessed for quality using the Qubit RNA Assay (Invitrogen, Carlsbad, CA, USA) and RNA ScreenTape analysis (TapeStation, Agilent Technologies Santa Clara, CA, USA). For mRNA enrichment, poly(A) mRNA was isolated using oligo(dT) magnetic beads. Library preparation for high-throughput sequencing followed the protocols provided in the NEBNext Ultra II RNA Library Prep Kit for Illumina (New England BioLabs, Ipswich, MA, USA), starting with approximately 250 ng of total RNA. Adapter-ligated, double-stranded cDNA fragments were then PCR-amplified for 12 cycles, incorporating Illumina P5/P7 adapters and unique barcode sequences. These uniquely barcoded libraries were pooled and sequenced on an Illumina HiSeq platform as per the manufacturer’s guidelines (Illumina, San Diego, CA, USA), employing a 150-bp paired-end configuration.

### Analysis of gene expression

The reference (AaegL5) gene and genome sequences^44^ and corresponding annotations were downloaded from Ensembl (http://metazoa.ensembl.org/Aedes_aegypti_lvpagwg/Info/Index). Raw reads were quality controlled and filtered with FastqMcf^79^. The clean reads were mapped to the gene reference using STAR^80^ with default parameters. Gene counts were computed using HT-Seq (htseq-count)^81^. The differential expression of genes was calculated using the edgeR package^82^ in R software (http://www.r-project.org/), with Benjamini–Hochberg adjusted P-values of 0.05 considered to be significant. GO-term enrichment analysis was performed using the Gene Ontology Enrichment tool from ShinyGO 0.80^83^ with default parameters (False Discovery Rate (FDR) cutoff: 0.05; number of pathways to show: 20; Min. and Max. pathway size: 2 and 2000) in term of biological process. Gene expression heatmaps were generated via ComplexHeatmap package^84^ and the principal component analysis plot was generated using BiocGenerics package^81^ in R.

### Clustering of genes based on expression patterns

Obtained transcriptome profiles of carcasses and reproductive organs in adult males and females of *Ae. aegypti* and *Ae. mascarensis* were clustered into distinct gene groups associated with expression patterns biased towards, or specific to, sex, tissue, and species. Using our pipeline (Supplementary Fig. 5a), we analyzed gene expression differences associated with species, sexes, and tissues in both pure species and abnormal hybrid males. To analyze gene expression linked to sex and tissue, we made five expression clusters using only pure species samples (see Supplementary Fig. 5b for sample usage): (1) Sex-specific/biased expression in reproductive organs (REPRODUCTIVE_SEX); (2) Species-specific/biased expression in reproductive organs (REPRODUCTIVE_SPECIES); (3) Sex-specific/biased expression in carcass (CARCASS_SEX); (4) Species-specific/biased expression in carcass (CARCASS_SPECIES); and (5) Tissue-specific/biased expression (TISSUE). Information about gene presence or absence in each cluster (‘1’ for presence or ‘0’ for absence) and gene numbers for each cluster can be found in Supplementary Data 5. A gene was considered in the ‘Low expression’ category if its FPKM was below 1 in both compared clusters of samples. If the FPKM value was more than 1 and there was no significant differential expression (FDR > 0.05) in a comparison, we considered a gene to be in the ‘Unbiased expression’ category. A threshold of a log twofold change (log_2_FC) = 2 was used to define unbiased expression: if log_2_FC ≤ 2, it was classified as unbiased expression. We assessed genes with log_2_FC > 2: if the FPKM value was less than 1 in one group, these genes were placed in the ‘Specific expression’ category; otherwise, genes were classified as the ‘Biased expression’ category (Supplementary Fig. 5a).

### Comparisons for differential gene expression in intersexes

Using created lists of genes based on the pattern of their expression (Supplementary Data 5 and 6), we further analyzed gene expression in five comparisons: U (*Ae. aegypti* Uganda) vs. UUM (normal backcrosses), M (*Ae. mascarensis*) vs. UUM, UUM vs. UUMix (intersexual backcrosses), U vs. UUMix, and M vs. UUMix. We focused our analysis on six groups of genes associated with sex or tissue (Supplementary Fig. 7): sex-specific/tissue-specific, sex-specific/tissue-biased, sex-specific/tissue-unbiased, sex-biased/tissue-specific, sex-biased/tissue-biased, and sex-biased/tissue-unbiased. To simplify the analysis, we did not consider out-group categories in a given sample, e.g. carcass-specific/-biased genes in reproductive organ samples or genes with low expression levels. To define expression patterns, thresholds of log_2_FC = 2 and FDR = 0.05 were used. Differential expression types were categorized as follows: (1) no change: −2 < log_2_FC < 2 with any FDR, or |log_2_FC | ≥ 2 with FDR >0.05; (2) upregulation: log_2_FC ≥ 2 with FDR ≤ 0.05; and (3) downregulation: log_2_FC ≤ 2 with FDR ≤ 0.05; and (4) other. First, we compared gene expression patterns between pure species and normal backcrosses (U vs. UUM and M vs. UUM). If the pattern deviated from ‘No change’, the gene was labeled as ‘Other’; otherwise, the gene was further compared between intersexual individuals and pure species or normal backcrosses (UUM vs. UUMix, U vs. UUMix, and M vs. UUMix). When compare UUM vs. UUMix, U vs. UUMix, and M vs. UUMix, expression patterns in all three comparisons had to be consistent for a gene to be classified as ‘No change’, ‘Downregulation’, and ‘Upregulation’; otherwise, it was labeled as ‘Other’.

### Statistics and reproducibility

All data were collected from three independent experiments unless otherwise stated. Statistical analysis of biological experiments was conducted using JMP Pro 16 (SAS Institute, Cary, NC, USA), The edgeR package^82^ in R software (http://www.r-project.org/) was employed for statistical analysis of the differential expression of genes. *P* values were calculated using the likelihood ratio test and Pearson test or using the Benjamini–Hochberg procedure. A *P* value of ≤0.05 was considered statistically significant. Details of the sample sizes and statistical analyses for each experiment are provided in ‘Results’ and ‘Methods’.

### Supplementary information

Supplementary Information

Description of Additional Supplementary Files

Supplementary Data 1-14

Supplementary Movie 1

## Data Availability

The RNA-seq data for this paper are available online at NCBI Sequence Read Archive (accession number: PRJNA1037487). All FPKM data and differential expression data are submitted with the manuscript.
