# Supplementary material for: Hybridization between Aedes aegypti and Aedes mascarensis mosquitoes leads to disruption of male sex determination
Source: Commun Biol. 2024 Jul 22;7:886. doi: 10.1038/s42003-024-06560-4 (PMC11263339; doi:10.1038/s42003-024-06560-4)
Supplement: Supplementary file 1 — Supplementary Information [file 42003_2024_6560_MOESM1_ESM.pdf]

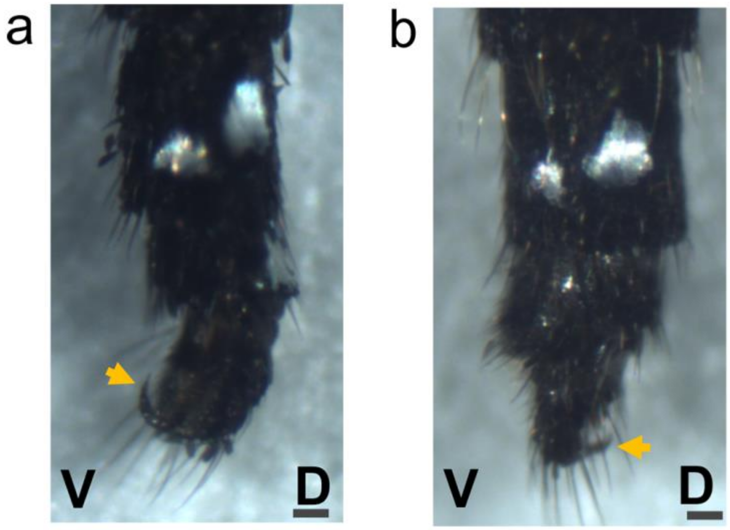

Supplementary Figure 1. A lateral view of the abdominal segments of backcrossed adult males. (a) Successful 180° rotation of the VIIIth abdominal segment. (b) Failed 180° rotation of the VIIIth abdominal segment. V, Ventral; D, Dorsal. Arrows point to the position of male clasper. Scale bars, 0.1 mm.

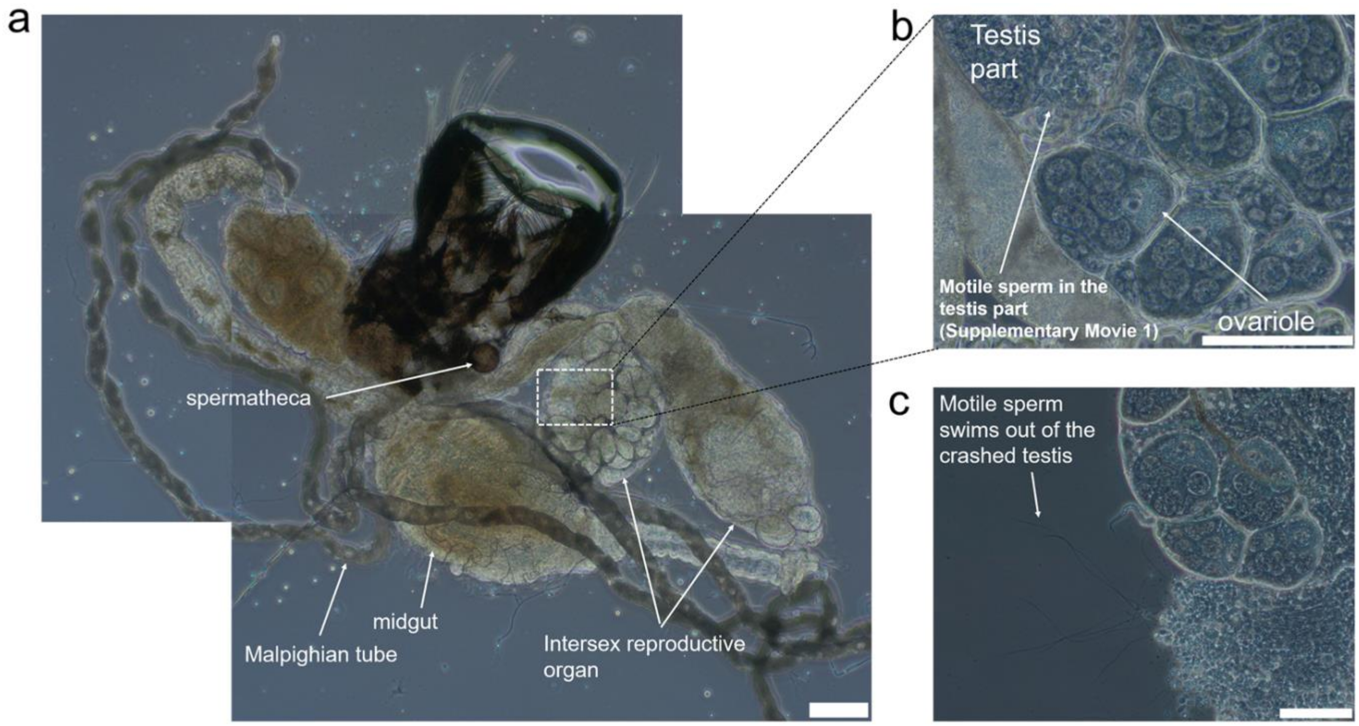

Supplementary Figure 2. Motile sperm, spermatheca, and mature ovarioles in the reproductive organ of an abnormal male. (a) The whole reproductive organ of the abnormal male. (b) Zoomed view of the reproductive tissue with both motile sperm and mature ovarioles, which corresponds to Supplementary Movie 1. (c) Motile sperm from the testis part of the crushed reproductive organ of the abnormal male. Scale bars, 100 µm.

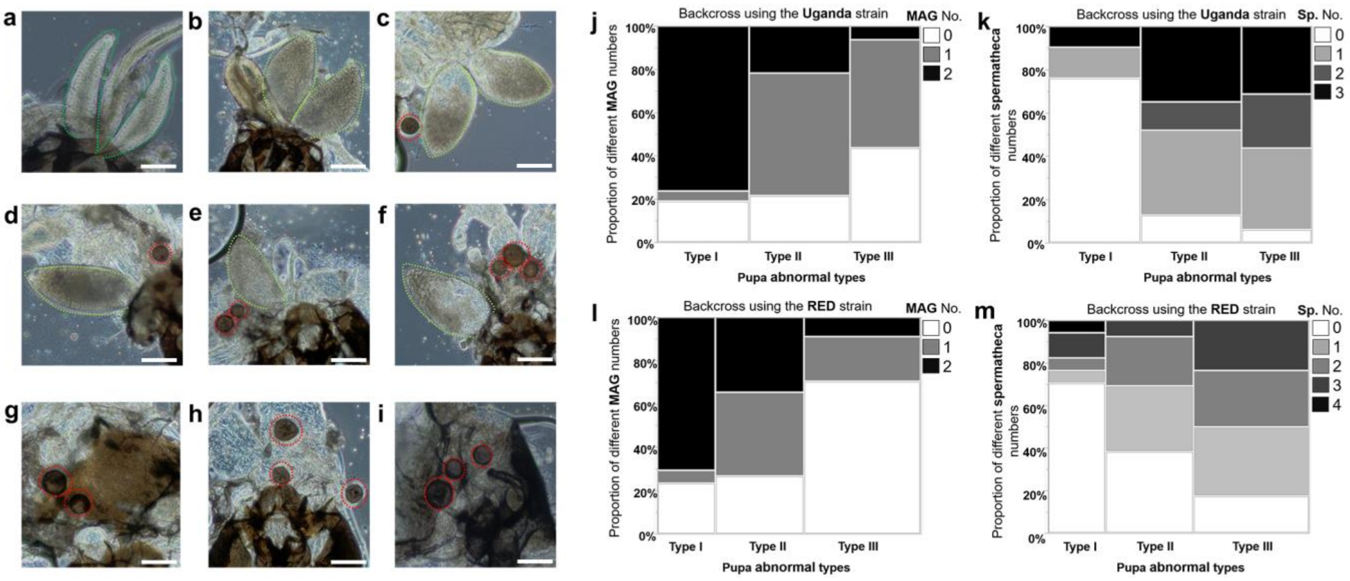

Supplementary Figure 3. Morphologies of somatic parts of reproductive organs in adults emerged from normal and abnormal male pupae. (a) A pair of MAGs in a normal male. (b) A pair of degenerated MAGs in an abnormal male. (c-f) Co-occurrence of degenerated MAGs and various numbers of spermathecae in abnormal males. (g-i) Absence of MAGs and presence of different numbers of spermathecae in abnormal males. (j) A mosaic plot showing the association between the number of MAGs in abnormal adults and the type of their pupae in the backcross between *Ae. aegypti* Uganda and *Ae. mascarensis* (UUM). Likelihood Ratio Test, P<0.0001; Pearson Test, P<0.0001; n=60. (k) A mosaic plot showing the association between the number of spermathecae in abnormal adults and the type of their pupae in backcrosses between *Ae. aegypti* Uganda and *Ae. mascarensis* (UUM). Likelihood Ratio Test, P<0.0001; Pearson Test, P<0.0001; n=60. (l) A mosaic plot showing the association between the number of MAGs in abnormal adults and the type of their pupae in backcrosses between *Ae. aegypti* RED and *Ae. mascarensis* (RRM). Likelihood Ratio Test, P<0.0001; Pearson Test, P<0.0001; n=77. (m) A mosaic plot showing the association between the number of spermatheca in abnormal adults and the type of their pupae in backcrosses between *Ae. aegypti* RED and *Ae. mascarensis* (RRM). Likelihood Ratio Test, P=0.0055; Pearson Test, P=0.0080; n=77. Green dots show the positions of MAGs and red dots show the positions of spermathecae. MAG, male accessary glands; Sp., spermathecae. Scale bars, 100 µm.

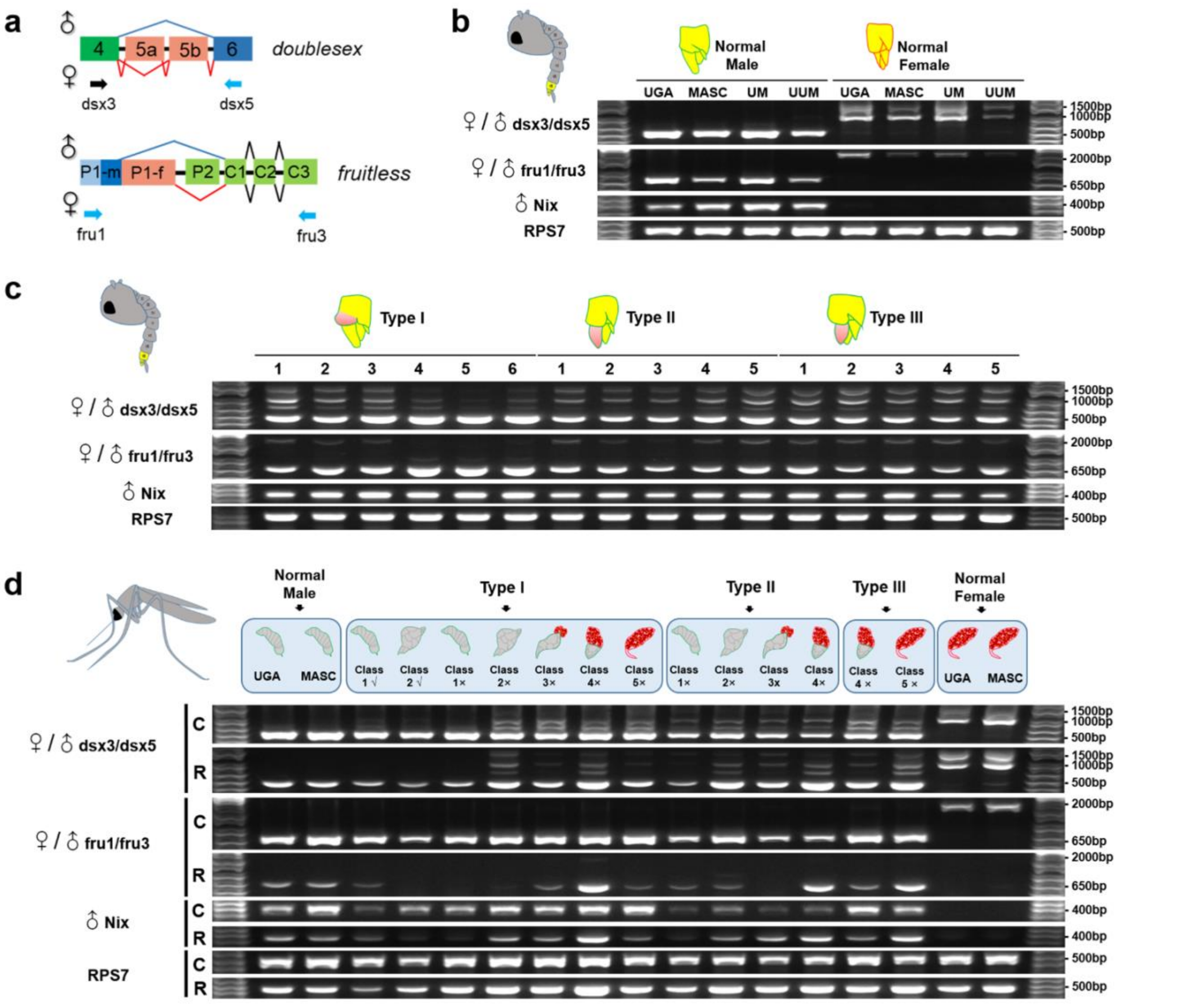

Supplementary Figure 4. RT-PCR of *doublesex* and *fruitless* male and female transcripts in pupae and adults of normal males and females, as well as abnormal males. (a) A scheme showing position of primers for both female and male transcripts of *dsx* and *fru* genes. (b) RT-PCR results obtained on normal male and female pupae from pure species, F1 hybrids, and backcrosses. (c) RT-PCR results obtained on abnormal male pupae of Type I, Type II, and Type III. Numbers in each type indicate different individuals. (d) RT-PCR results obtained on abnormal adults emerged from different types of pupae. *Nix* was used as a positive control for male-specific expression. *RPS7* was used as an endogenous control gene. UGA, *Ae. aeypti* Uganda strain; MASC, *Ae. mascarensis*; UM, F1 hybrid between female UGA and male MASC; UUM, backrossing generations from female UGA and male F1UM; √/×, successful/failed 180° rotation of VIIIth abdominal segment; C, carcass; R, reproductive organs.

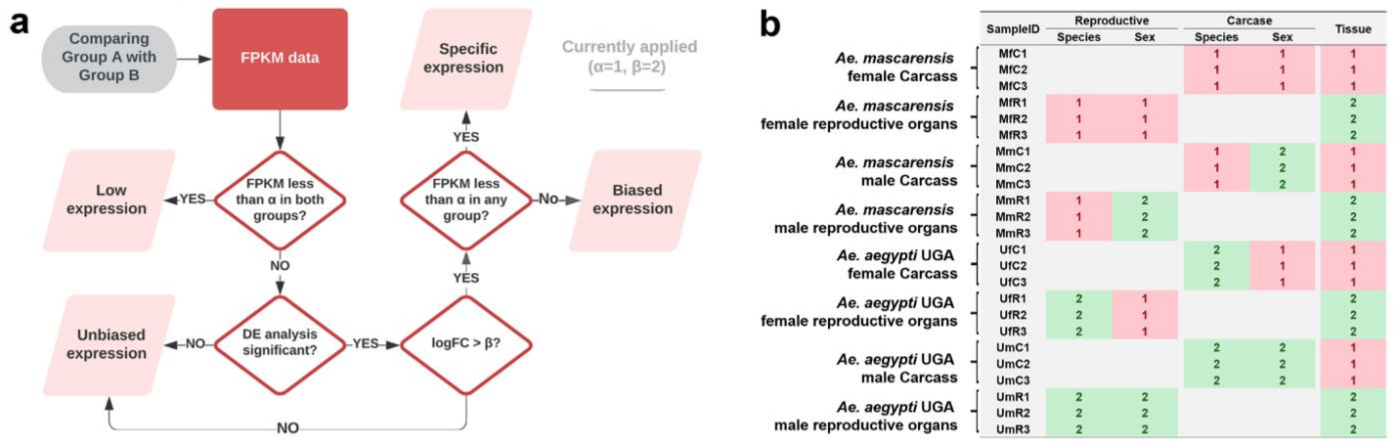

Supplementary Figure 5. Pipeline of grouping genes using RNA-seq data from *Ae. aegypti* and *Ae. mascarensis*. (a) Pipeline for identifying expression patterns. (b) Sample usage for differential expression comparisons. Each column represents a specific comparison, with the samples utilized in that comparison indicated. The markers ('1' or '2') denote the distinct groups within each comparison.

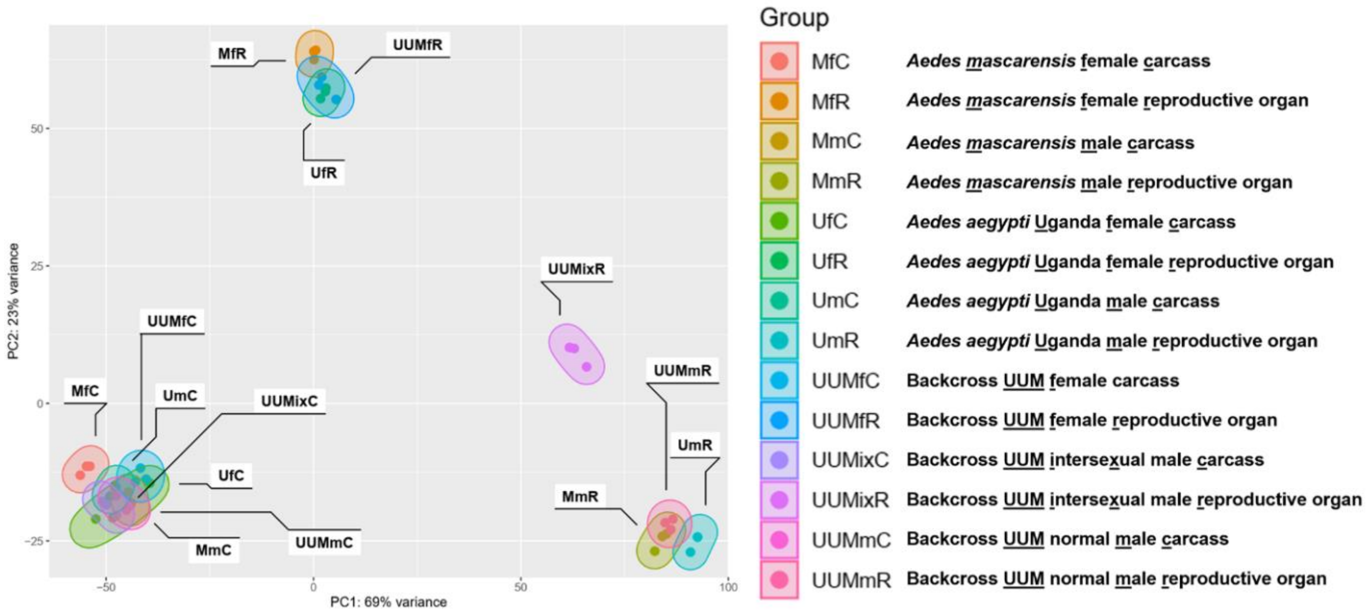

Supplementary Figure 6. Principal component analysis on the RNA-seq data from all samples.

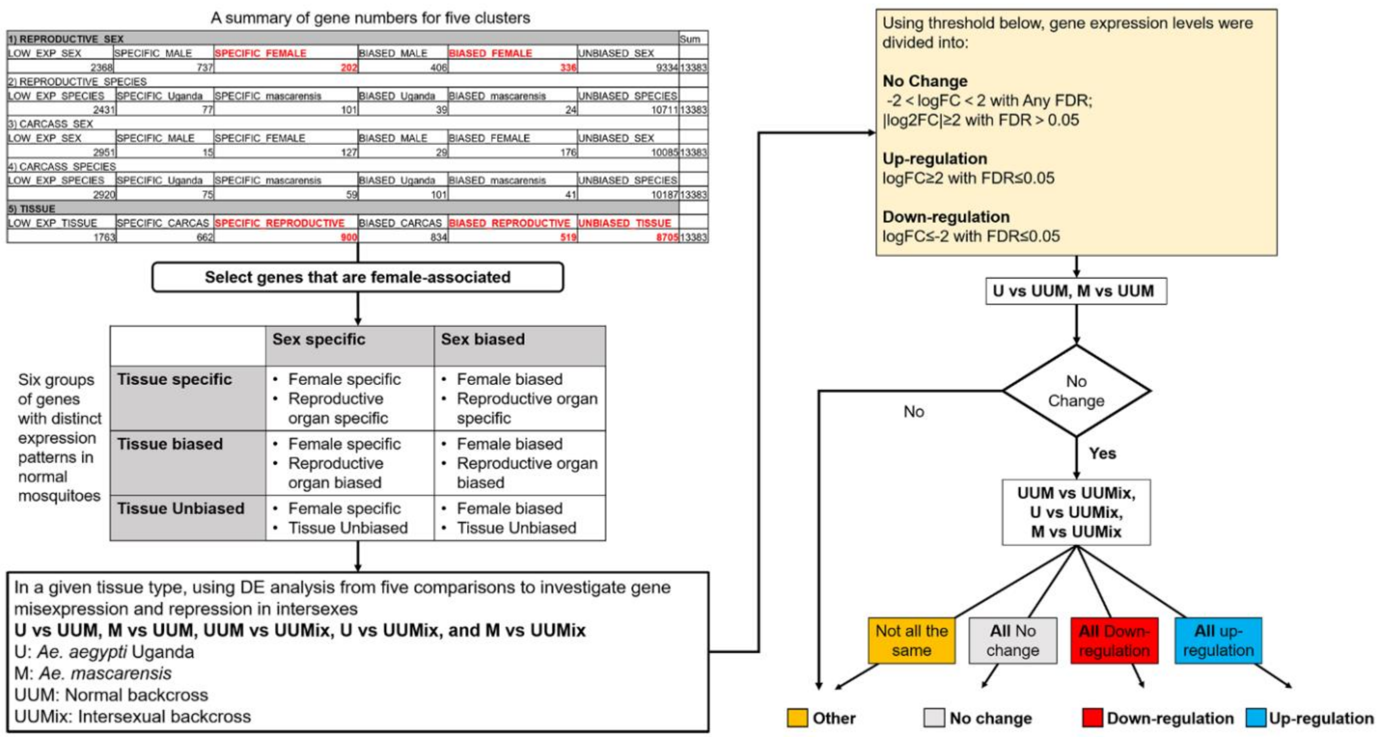

Supplementary Figure 7. Pipeline to analyze gene expression in intersexes using data from female reproductive organs as an example.

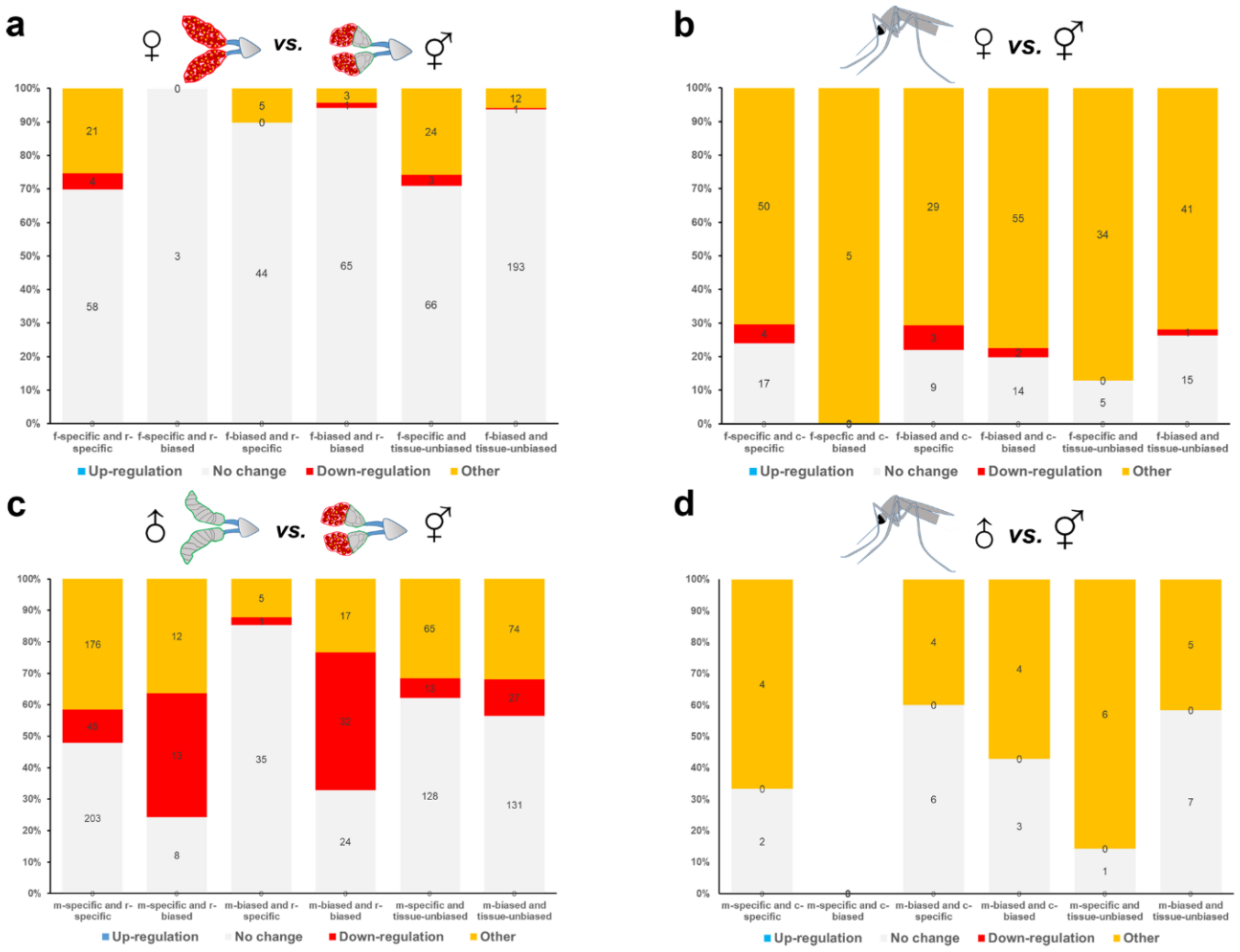

Supplementary Figure 8. A summary of gene differential expression between intersexes and males or females. (a) Gene numbers and percentages of six groups of genes in reproductive organs of intersexes compared with females. (b) Gene numbers and percentages of six groups of genes in carcasses of intersexes compared with females. (c) Gene numbers and percentages of six groups of genes in reproductive organs of intersexes compared with normal males. (d) Gene numbers and percentages of six groups of genes in carcasses of intersex males compared with normal males. f, female; m, male, r, reproductive organ, c, carcass.

a

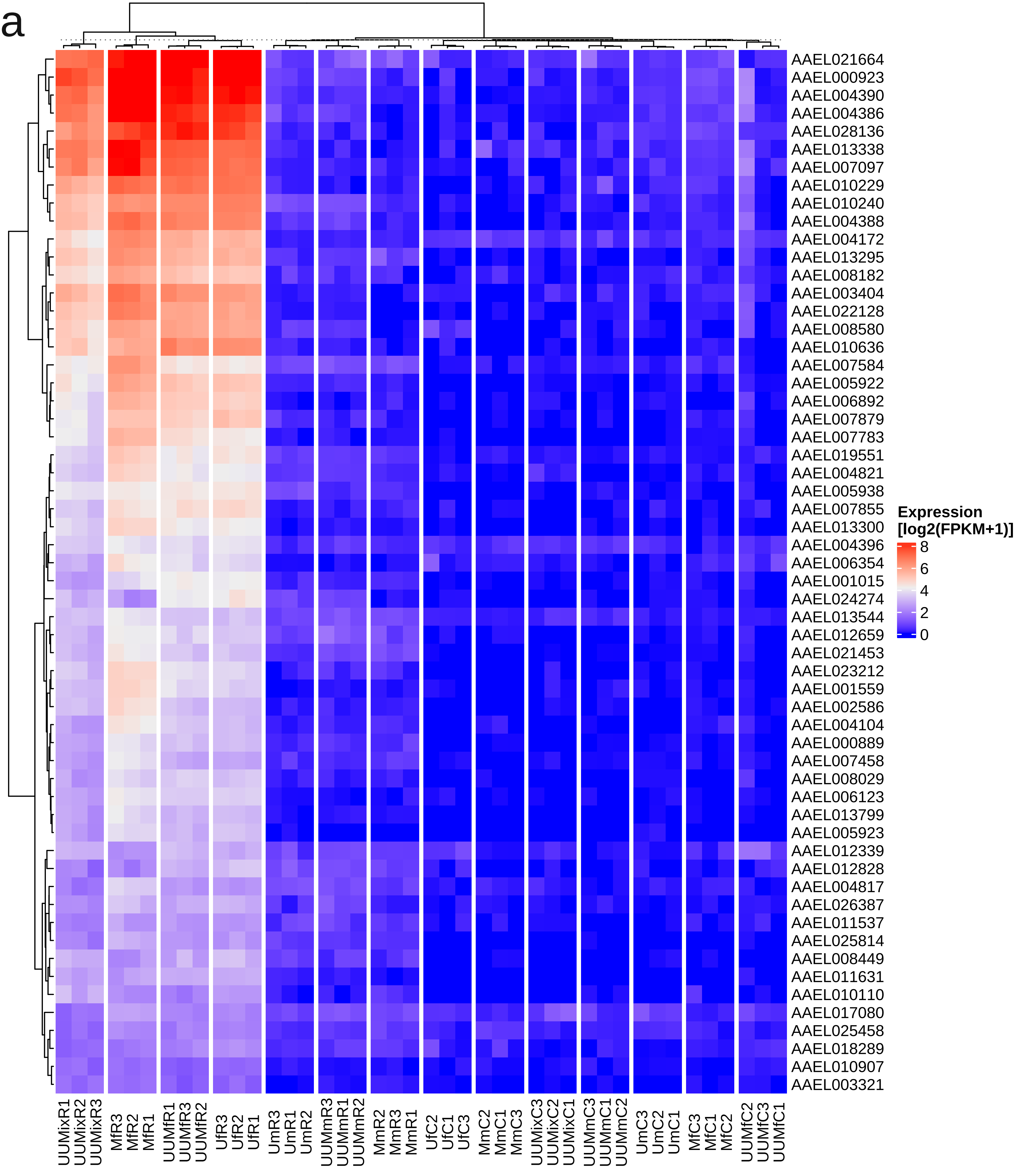
AAEL021664
AAEL000923
AAEL004390
AAEL004386
AAEL028136
AAEL013338
AAEL007097
AAEL010229
AAEL010240
AAEL004388
AAEL004172
AAEL013295
AAEL008182
AAEL003404
AAEL022128
AAEL008580
AAEL010636
AAEL007584
AAEL005922
AAEL006892
AAEL007879
AAEL007783
AAEL019551
AAEL004821
AAEL005938
AAEL007855
AAEL013300
AAEL004396
AAEL006354
AAEL001015
AAEL024274
AAEL013544
AAEL012659
AAEL021453
AAEL023212
AAEL001559
AAEL002586
AAEL004104
AAEL000889
AAEL007458
AAEL008029
AAEL006123
AAEL013799
AAEL005923
AAEL012339
AAEL012828
AAEL004817
AAEL026387
AAEL011537
AAEL025814
AAEL008449
AAEL011631
AAEL010110
AAEL017080
AAEL025458
AAEL018289
AAEL010907
AAEL003321
Expression
[log2(FPKM+1)]
8
6
4
2
0
UUMixR1 UUMixR2 UUMixR3
MfR3 MfR2 MfR1
UUMfR1 UUMfR3 UUMfR2
UfR3 UfR2 UfR1
UmR3 UmR1 UmR2
UUMmR3 UUMmR1 UUMmR2
MmR2 MmR3 MmR1
UfC2 UfC1 UfC3
MmC2 MmC1 MmC3
UUMixC3 UUMixC2 UUMixC1
UUMmC3 UUMmC1 UUMmC2
UmC3 UmC2 UmC1
MfC3 MfC1 MfC2
UUMfC2 UUMfC3 UUMfC1

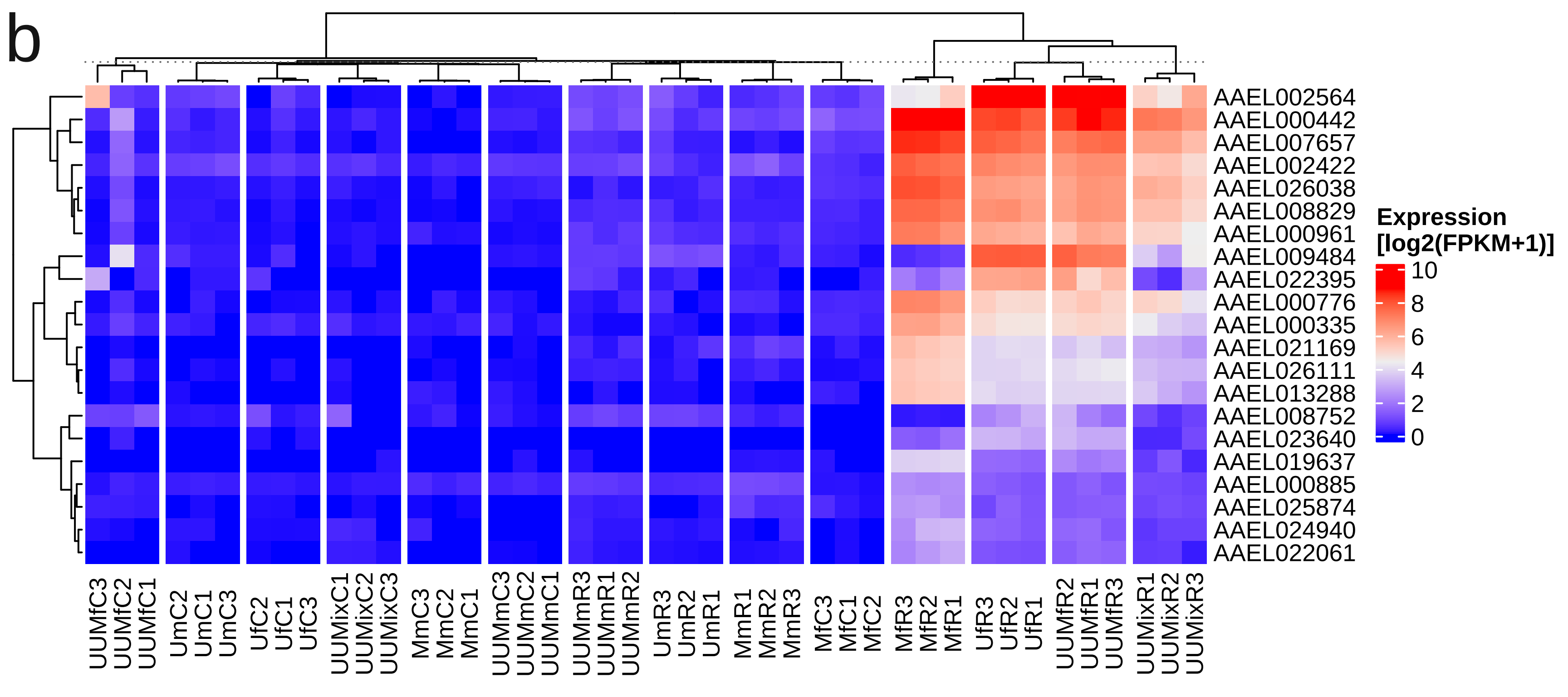
b
AAEL002564
AAEL000442
AAEL007657
AAEL002422
AAEL026038
AAEL008829
AAEL000961
AAEL009484
AAEL022395
AAEL000776
AAEL000335
AAEL021169
AAEL026111
AAEL013288
AAEL008752
AAEL023640
AAEL019637
AAEL000885
AAEL025874
AAEL024940
AAEL022061
Expression
[log2(FPKM+1)]
10
8
6
4
2
0
UUMfC3
UUMfC2
UUMfC1
UmC2
UmC1
UmC3
UfC2
UfC1
UfC3
UUMixC1
UUMixC2
UUMixC3
MmC3
MmC2
MmC1
UUMmC3
UUMmC2
UUMmC1
UUMmR3
UUMmR1
UUMmR2
UmR3
UmR2
UmR1
MmR1
MmR2
MmR3
MfC3
MfC1
MfC2
MfR3
MfR2
MfR1
UfR3
UfR2
UfR1
UUMfR2
UUMfR1
UUMfR3
UUMixR1
UUMixR2
UUMixR3

C

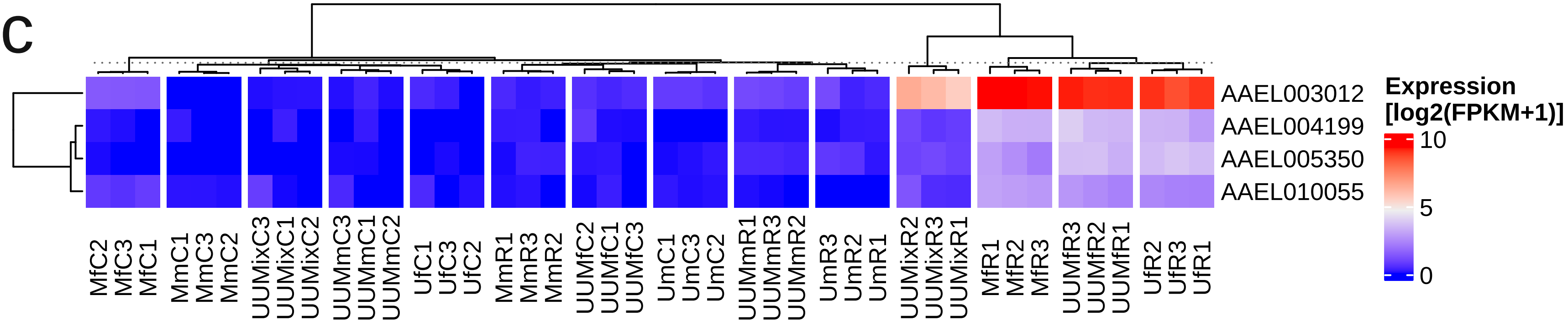

AAEL003012
AAEL004199
AAEL005350
AAEL010055
Expression [log2(FPKM+1)]
10
5
0
MfC2 MfC3 MfC1 MmC1 MmC3 MmC2 UUMixC3 UUMixC1 UUMixC2 UUMmC3 UUMmC1 UUMmC2 UfC1 UfC3 UfC2 MmR1 MmR3 MmR2 UUMfC2 UUMfC1 UUMfC3 UmC1 UmC3 UmC2 UUMmR1 UUMmR3 UUMmR2 UmR3 UmR2 UmR1 UUMixR2 UUMixR3 UUMixR1 MfR1 MfR2 MfR3 UUMfR3 UUMfR2 UUMfR1 UfR2 UfR3 UfR1

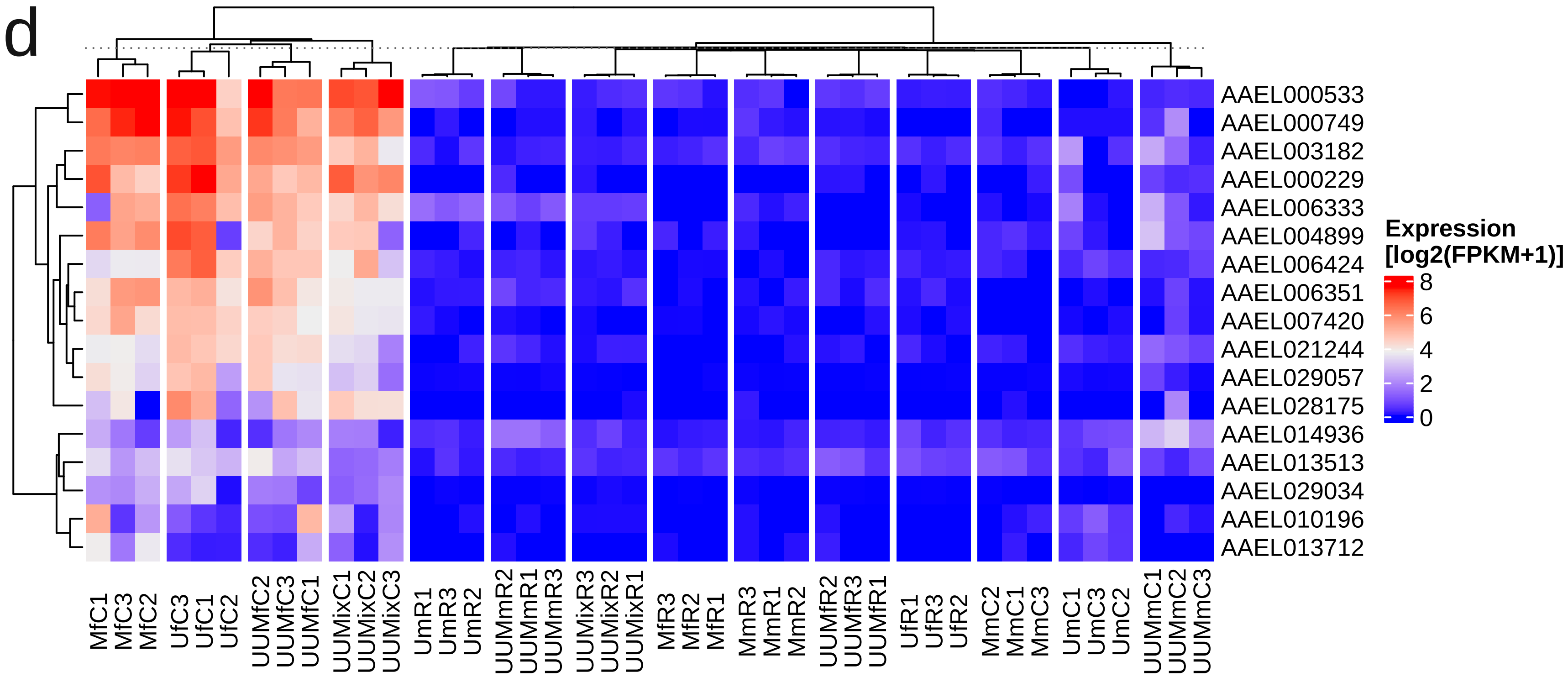
d
AAEL000533
AAEL000749
AAEL003182
AAEL000229
AAEL006333
AAEL004899
AAEL006424
AAEL006351
AAEL007420
AAEL021244
AAEL029057
AAEL028175
AAEL014936
AAEL013513
AAEL029034
AAEL010196
AAEL013712
Expression
[log2(FPKM+1)]
8
6
4
2
0
MfC1
MfC3
MfC2
UfC3
UfC1
UfC2
UUMfC2
UUMfC3
UUMfC1
UUMixC1
UUMixC2
UUMixC3
UmR1
UmR3
UmR2
UUMmR2
UUMmR1
UUMmR3
UUMixR3
UUMixR2
UUMixR1
MfR3
MfR2
MfR1
MmR3
MmR1
MmR2
UUMfR2
UUMfR3
UUMfR1
UfR1
UfR3
UfR2
MmC2
MmC1
MmC3
UmC1
UmC3
UmC2
UUMmC1
UUMmC2
UUMmC3

e

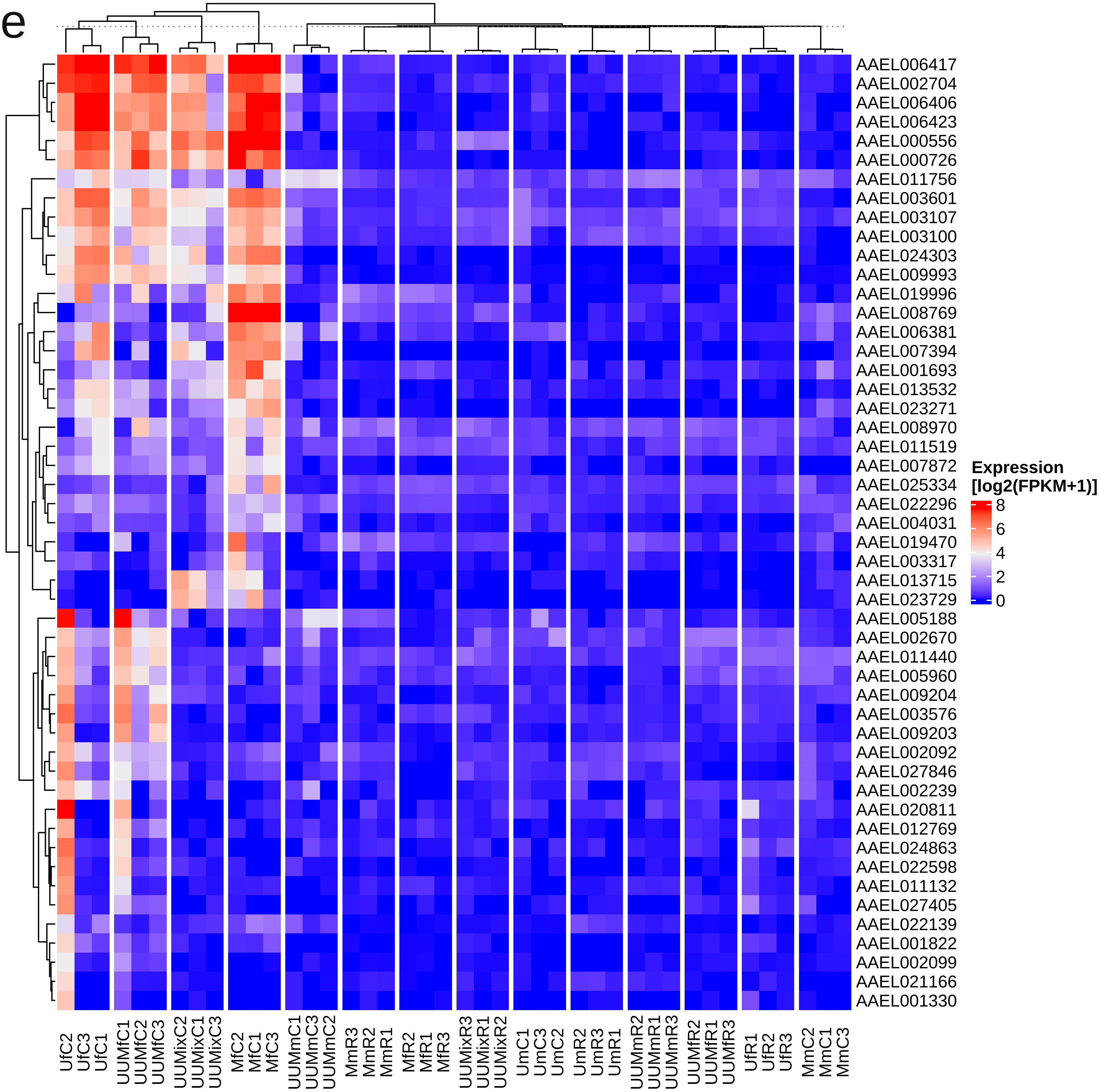
AAEL006417
AAEL002704
AAEL006406
AAEL006423
AAEL000556
AAEL000726
AAEL011756
AAEL003601
AAEL003107
AAEL003100
AAEL024303
AAEL009993
AAEL019996
AAEL008769
AAEL006381
AAEL007394
AAEL001693
AAEL013532
AAEL023271
AAEL008970
AAEL011519
AAEL007872
AAEL025334
AAEL022296
AAEL004031
AAEL019470
AAEL003317
AAEL013715
AAEL023729
AAEL005188
AAEL002670
AAEL011440
AAEL005960
AAEL009204
AAEL003576
AAEL009203
AAEL002092
AAEL027846
AAEL002239
AAEL020811
AAEL012769
AAEL024863
AAEL022598
AAEL011132
AAEL027405
AAEL022139
AAEL001822
AAEL002099
AAEL021166
AAEL001330
Expression
[log2(FPKM+1)]
8
6
4
2
0
UfC2
UfC3
UfC1
UUMfC1
UUMfC2
UUMfC3
UUMixC2
UUMixC1
UUMixC3
MfC2
MfC1
MfC3
UUMmC1
UUMmC3
UUMmC2
MmR3
MmR2
MmR1
MfR2
MfR1
MfR3
UUMixR3
UUMixR1
UUMixR2
UmC1
UmC3
UmC2
UmR2
UmR3
UmR1
UUMmR2
UUMmR1
UUMmR3
UUMfR2
UUMfR1
UUMfR3
UfR1
UfR2
UfR3
MmC2
MmC1
MmC3

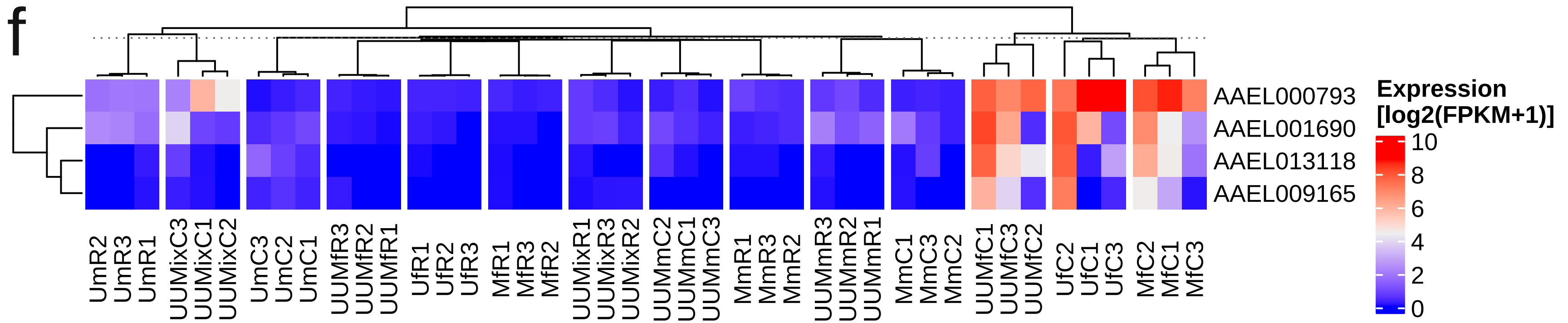
f
AAEL000793
AAEL001690
AAEL013118
AAEL009165
Expression
[log2(FPKM+1)]
10
8
6
4
2
0
UmR2
UmR3
UmR1
UUMixC3
UUMixC1
UUMixC2
UmC3
UmC2
UmC1
UUMfR3
UUMfR2
UUMfR1
UfR1
UfR2
UfR3
MfR1
MfR3
MfR2
UUMixR1
UUMixR3
UUMixR2
UUMmC2
UUMmC1
UUMmC3
MmR1
MmR3
MmR2
UUMmR3
UUMmR2
UUMmR1
MmC1
MmC3
MmC2
UUMfC1
UUMfC3
UUMfC2
UfC2
UfC1
UfC3
MfC2
MfC1
MfC3

g

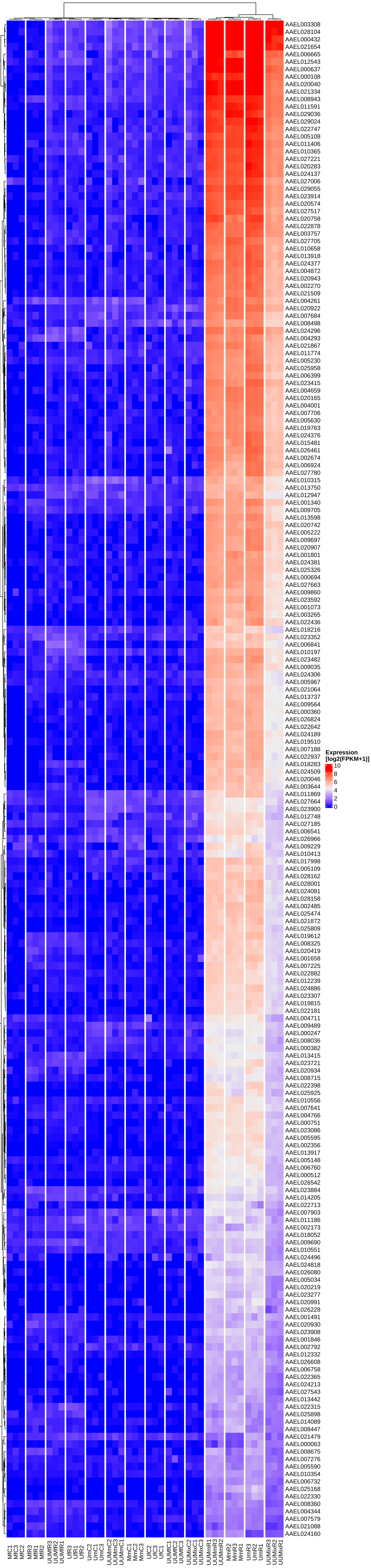

AAEL003308
AAEL028104
AAEL000432
AAEL021654
AAEL006665
AAEL012543
AAEL000637
AAEL000108
AAEL020040
AAEL021334
AAEL008943
AAEL011591
AAEL029036
AAEL029024
AAEL022747
AAEL005108
AAEL011406
AAEL010365
AAEL027221
AAEL020283
AAEL024137
AAEL027006
AAEL029055
AAEL023914
AAEL020574
AAEL027517
AAEL020758
AAEL022878
AAEL003757
AAEL027705
AAEL010658
AAEL013918
AAEL024377
AAEL004872
AAEL020943
AAEL002270
AAEL021509
AAEL004261
AAEL020922
AAEL007684
AAEL008498
AAEL024296
AAEL004293
AAEL021867
AAEL011774
AAEL005230
AAEL025958
AAEL006399
AAEL023415
AAEL004659
AAEL020165
AAEL004001
AAEL007706
AAEL005630
AAEL019763
AAEL024376
AAEL015481
AAEL026461
AAEL002674
AAEL006924
AAEL027780
AAEL010315
AAEL013750
AAEL012947
AAEL001340
AAEL009705
AAEL013598
AAEL020742
AAEL005222
AAEL009697
AAEL020907
AAEL001801
AAEL024381
AAEL025326
AAEL000694
AAEL027663
AAEL009860
AAEL023592
AAEL001073
AAEL003265
AAEL022436
AAEL018216
AAEL023352
AAEL006841
AAEL010197
AAEL023482
AAEL009035
AAEL024306
AAEL005967
AAEL021064
AAEL013737
AAEL009564
AAEL000360
AAEL026824
AAEL022642
AAEL024189
AAEL019510
AAEL007188
AAEL022937
AAEL018283
AAEL024509
AAEL020046
AAEL003644
AAEL011869
AAEL027664
AAEL023900
AAEL012748
AAEL027185
AAEL006541
AAEL026966
AAEL009229
AAEL010413
AAEL017998
AAEL005109
AAEL028162
AAEL028001
AAEL024081
AAEL028158
AAEL002485
AAEL025474
AAEL021872
AAEL025809
AAEL019612
AAEL008325
AAEL020419
AAEL001658
AAEL007225
AAEL022882
AAEL012239
AAEL024886
AAEL023307
AAEL019815
AAEL022181
AAEL004711
AAEL009489
AAEL000247
AAEL008036
AAEL000382
AAEL013415
AAEL023721
AAEL020934
AAEL008715
AAEL022398
AAEL025925
AAEL010556
AAEL007641
AAEL004766
AAEL000751
AAEL023086
AAEL005595
AAEL002356
AAEL013917
AAEL005148
AAEL006760
AAEL000512
AAEL026542
AAEL023884
AAEL014205
AAEL022713
AAEL007903
AAEL011186
AAEL002173
AAEL018052
AAEL009690
AAEL010551
AAEL024496
AAEL024818
AAEL026080
AAEL005034
AAEL020219
AAEL023277
AAEL020991
AAEL026228
AAEL001491
AAEL020930
AAEL023908
AAEL001846
AAEL002792
AAEL012332
AAEL026608
AAEL006758
AAEL022365
AAEL024213
AAEL027543
AAEL013442
AAEL022315
AAEL025898
AAEL014089
AAEL008447
AAEL021479
AAEL000063
AAEL008675
AAEL007276
AAEL005590
AAEL010354
AAEL006732
AAEL025168
AAEL022330
AAEL008360
AAEL004344
AAEL007579
AAEL021088
AAEL024160
Expression
[log2(FPKM+1)]
10
8
6
4
2
0
MfC1
MfC3
MfC2
MfR3
MfR1
MfR2
UUMfR3
UUMfR2
UUMfR1
UfR3
UfR1
UfR2
UmC2
UmC1
UmC3
UUMmC2
UUMmC3
UUMmC1
MmC1
MmC2
MmC3
UfC2
UfC3
UfC1
UUMfC1
UUMfC2
UUMfC3
UUMixC2
UUMixC1
UUMixC3
UUMmR1
UUMmR3
UUMmR2
MmR2
MmR3
MmR1
UmR3
UmR2
UmR1
UUMixR3
UUMixR2
UUMixR1

h

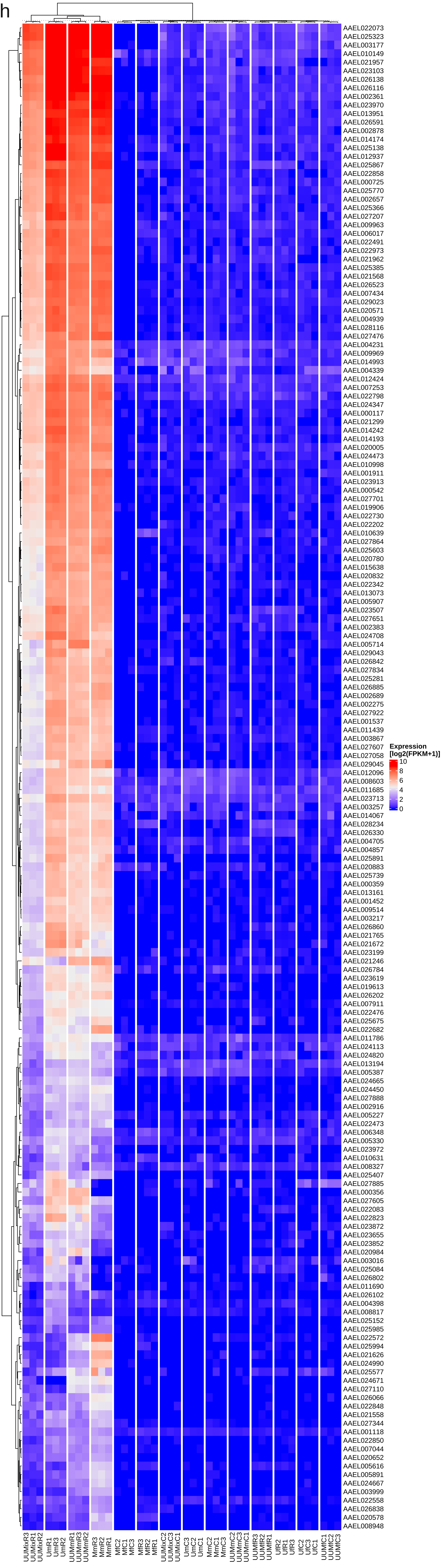

AAEL022073
AAEL025323
AAEL003177
AAEL010149
AAEL021957
AAEL023103
AAEL026138
AAEL026116
AAEL002361
AAEL023970
AAEL013951
AAEL026591
AAEL002878
AAEL014174
AAEL025138
AAEL012937
AAEL025867
AAEL022858
AAEL000725
AAEL025770
AAEL002657
AAEL025366
AAEL027207
AAEL009963
AAEL006017
AAEL022491
AAEL022973
AAEL021962
AAEL025385
AAEL021568
AAEL026523
AAEL007434
AAEL029023
AAEL020571
AAEL004939
AAEL028116
AAEL027476
AAEL004231
AAEL009969
AAEL014993
AAEL004339
AAEL012424
AAEL007253
AAEL022798
AAEL024347
AAEL000117
AAEL021299
AAEL014242
AAEL014193
AAEL020005
AAEL024473
AAEL010998
AAEL001911
AAEL023913
AAEL000542
AAEL027701
AAEL019906
AAEL022730
AAEL022202
AAEL010639
AAEL027864
AAEL025603
AAEL020780
AAEL015638
AAEL020832
AAEL022342
AAEL013073
AAEL005907
AAEL023507
AAEL027651
AAEL002383
AAEL024708
AAEL005714
AAEL029043
AAEL026842
AAEL027834
AAEL025281
AAEL026885
AAEL002689
AAEL002275
AAEL027922
AAEL001537
AAEL011439
AAEL003867
AAEL027607
AAEL027058
AAEL029045
AAEL012096
AAEL008603
AAEL011685
AAEL023713
AAEL003257
AAEL014067
AAEL028234
AAEL026330
AAEL004705
AAEL004857
AAEL025891
AAEL020883
AAEL025739
AAEL000359
AAEL013161
AAEL001452
AAEL009514
AAEL003217
AAEL026860
AAEL021765
AAEL021672
AAEL023199
AAEL021246
AAEL026784
AAEL023619
AAEL019613
AAEL026202
AAEL007911
AAEL022476
AAEL025675
AAEL022682
AAEL011786
AAEL024113
AAEL024820
AAEL013194
AAEL005387
AAEL024665
AAEL024450
AAEL027888
AAEL002916
AAEL005227
AAEL022473
AAEL006348
AAEL005330
AAEL023972
AAEL010631
AAEL008327
AAEL025407
AAEL027885
AAEL000356
AAEL027605
AAEL022083
AAEL022823
AAEL023872
AAEL023655
AAEL023852
AAEL020984
AAEL003016
AAEL025084
AAEL026802
AAEL011690
AAEL026102
AAEL004398
AAEL008817
AAEL025152
AAEL025985
AAEL022572
AAEL025994
AAEL021626
AAEL024990
AAEL025577
AAEL024671
AAEL027110
AAEL026066
AAEL022848
AAEL021558
AAEL027344
AAEL001118
AAEL022850
AAEL007044
AAEL020652
AAEL005616
AAEL005891
AAEL024667
AAEL003999
AAEL022558
AAEL026838
AAEL020578
AAEL008948
Expression
[log2(FPKM+1)]
10
8
6
4
2
0
UUMixR3
UUMixR1
UUMixR2
UmR1
UmR3
UmR2
UUMmR1
UUMmR3
UUMmR2
MmR3
MmR2
MmR1
MfC2
MfC1
MfC3
MfR3
MfR2
MfR1
UUMixC2
UUMixC3
UUMixC1
UmC3
UmC2
UmC1
MmC2
MmC1
MmC3
UUMmC2
UUMmC3
UUMmC1
UUMfR3
UUMfR2
UUMfR1
UfR2
UfR1
UfR3
UfC2
UfC3
UfC1
UUMfC1
UUMfC2
UUMfC3

i

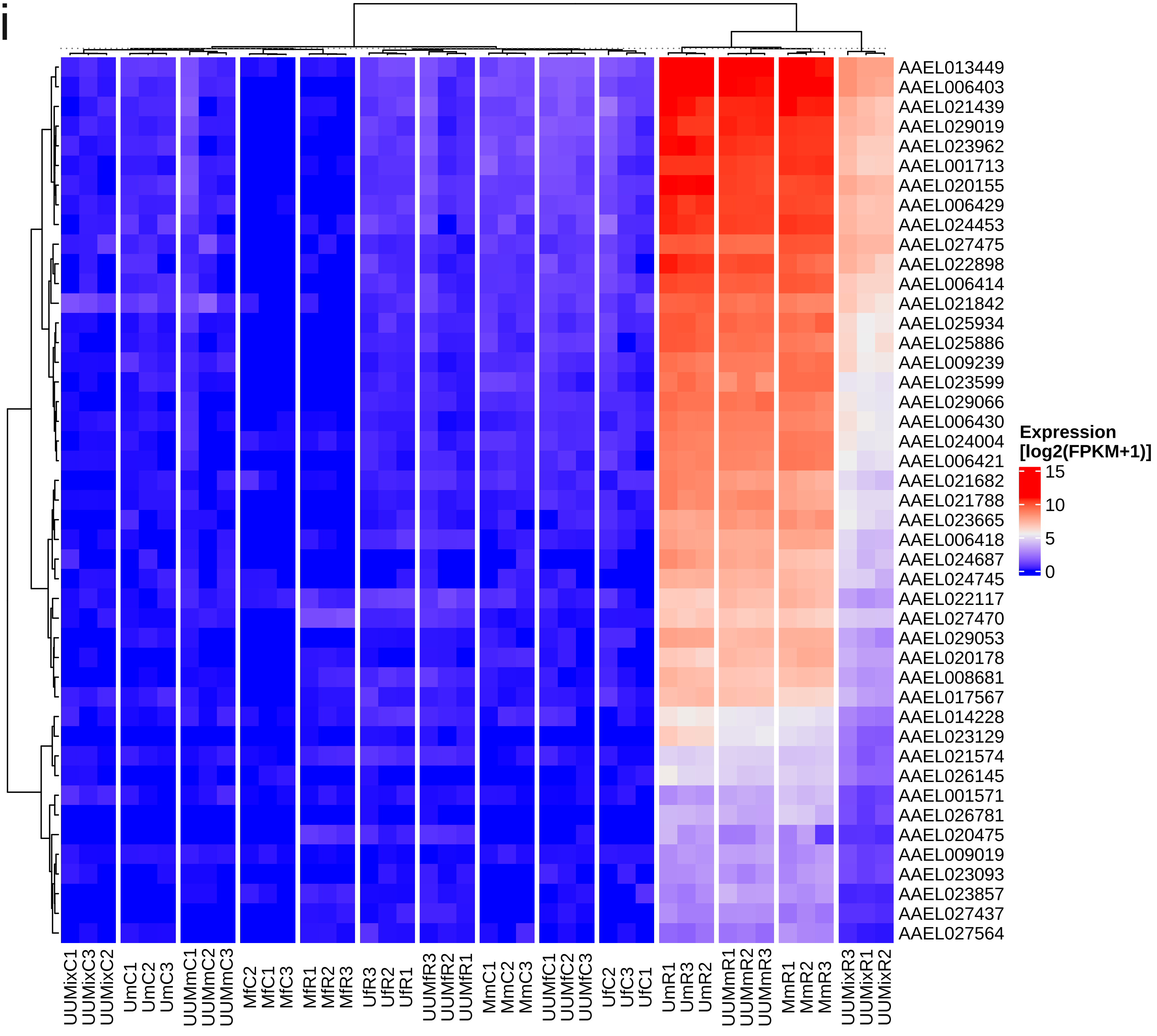

AAEL013449
AAEL006403
AAEL021439
AAEL029019
AAEL023962
AAEL001713
AAEL020155
AAEL006429
AAEL024453
AAEL027475
AAEL022898
AAEL006414
AAEL021842
AAEL025934
AAEL025886
AAEL009239
AAEL023599
AAEL029066
AAEL006430
AAEL024004
AAEL006421
AAEL021682
AAEL021788
AAEL023665
AAEL006418
AAEL024687
AAEL024745
AAEL022117
AAEL027470
AAEL029053
AAEL020178
AAEL008681
AAEL017567
AAEL014228
AAEL023129
AAEL021574
AAEL026145
AAEL001571
AAEL026781
AAEL020475
AAEL009019
AAEL023093
AAEL023857
AAEL027437
AAEL027564
Expression
[log2(FPKM+1)]
15
10
5
0
UUMixC1
UUMixC3
UUMixC2
UmC1
UmC2
UmC3
UUMmC1
UUMmC2
UUMmC3
MfC2
MfC1
MfC3
MfR1
MfR2
MfR3
UfR3
UfR2
UfR1
UUMfR3
UUMfR2
UUMfR1
MmC1
MmC2
MmC3
UUMfC1
UUMfC2
UUMfC3
UfC2
UfC3
UfC1
UmR1
UmR3
UmR2
UUMmR1
UUMmR2
UUMmR3
MmR1
MmR2
MmR3
UUMixR3
UUMixR1
UUMixR2

j

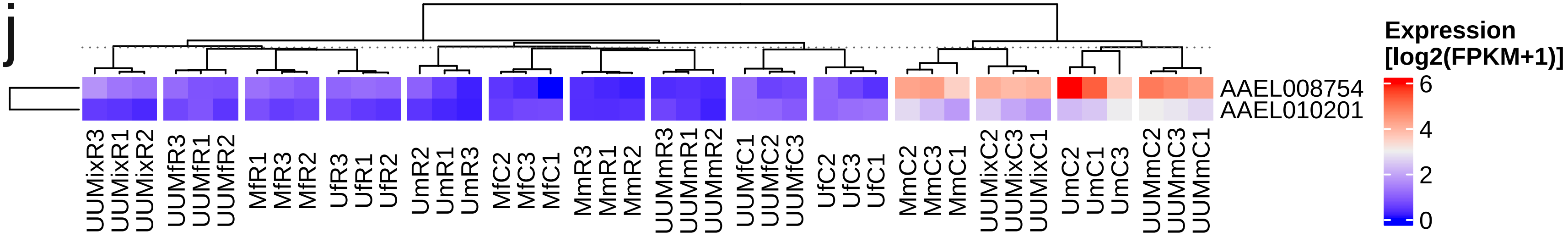

AAEL008754
AAEL010201
UUMixR3
UUMixR1
UUMixR2
UUMfR3
UUMfR1
UUMfR2
MfR1
MfR3
MfR2
UfR3
UfR1
UfR2
UmR2
UmR1
UmR3
MfC2
MfC3
MfC1
MmR3
MmR1
MmR2
UUMmR3
UUMmR1
UUMmR2
UUMfC1
UUMfC2
UUMfC3
UfC2
UfC3
UfC1
MmC2
MmC3
MmC1
UUMixC2
UUMixC3
UUMixC1
UmC2
UmC1
UmC3
UUMmC2
UUMmC3
UUMmC1
Expression
[log2(FPKM+1)]
6
4
2
0

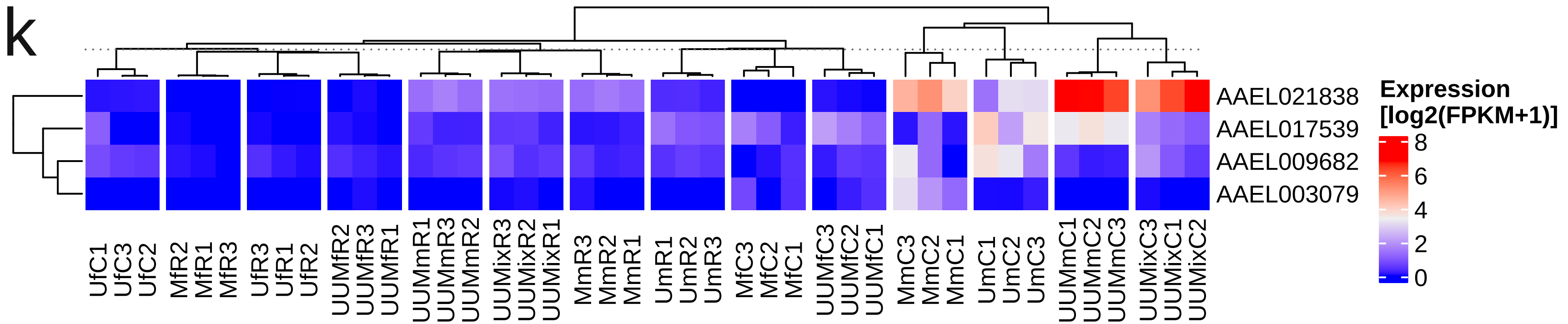
k
AAEL021838
AAEL017539
AAEL009682
AAEL003079
Expression
[log2(FPKM+1)]
8
6
4
2
0
UfC1
UfC3
UfC2
MfR2
MfR1
MfR3
UfR3
UfR1
UfR2
UUMfR2
UUMfR3
UUMfR1
UUMmR1
UUMmR3
UUMmR2
UUMixR3
UUMixR2
UUMixR1
MmR3
MmR2
MmR1
UmR1
UmR2
UmR3
MfC3
MfC2
MfC1
UUMfC3
UUMfC2
UUMfC1
MmC3
MmC2
MmC1
UmC1
UmC2
UmC3
UUMmC1
UUMmC2
UUMmC3
UUMixC3
UUMixC1
UUMixC2

Supplementary Figure 9. Expression heatmaps of sex-specific and carcass-specific genes in pure species and backcross individuals. (a) Female-specific/reproductive-specific gene list with ‘No change’ expression pattern in intersexes. (b) Female-specific/reproductive-specific gene list with ‘Other’ expression pattern in intersexes. (c) Female-specific/reproductive-specific gene list with ‘Down-regulation’ expression pattern in intersexes. (d) Female-specific/carcass-specific gene list with ‘No change’ expression pattern in intersexes. (e) Female-specific/carcass-specific gene list with ‘Other’ expression pattern in intersexes. (f) Female-specific/carcass-specific gene list with ‘Down-regulation’ expression pattern in intersexes. (g) Male-specific/reproductive-specific gene list with ‘No change’ expression pattern in intersexes. (h) Male-specific/reproductive-specific gene list with ‘Other’ expression pattern in intersexes. (i) Male-specific/reproductive-specific gene list with ‘Down-regulation’ expression pattern in intersexes. (j) Male-specific/carcass-specific gene list with ‘No change’ expression pattern in intersexes. (k) Male-specific/carcass-specific gene list with ‘Other’ expression pattern in intersexes.
