# Supplementary material for: Hybridization between Aedes aegypti and Aedes mascarensis mosquitoes leads to disruption of male sex determination
Source: Commun Biol. 2024 Jul 22;7:886. doi: 10.1038/s42003-024-06560-4 (PMC11263339; doi:10.1038/s42003-024-06560-4)
Supplement: Supplementary file 2 — Description of Additional Supplementary Files [file 42003_2024_6560_MOESM2_ESM.pdf]

Description of Additional Supplementary Files

File name: Supplementary Data 1

Description: Numbers of females, normal males, and abnormal males at the pupa stage in progenies from UUM and RRM crosses.

File name: Supplementary Data 2

Description: The morphology of abnormal male at the adult stage from the third progeny of the UUM backcross.

File name: Supplementary Data 3.

Description: The morphology of abnormal male at the adult stage from the five RRM backcrosses.

File name: Supplementary Data 4.
Description: Primer sequences for RT-PCR.

File name: Supplementary Data 5.

Description: Gene expression labels and a summary of gene numbers for each expression cluster based on RNA-seq data from pure species. (a) Gene expression labels. (b) A summary of gene numbers for each cluster. The pipeline is shown in Supplementary Figure 6. Numbers '1' and '0' in (a) mean gene presence and absence, respectively.

File name: Supplementary Data 6.
Description: Gene lists for female (f)- and reproductive (r)-associated expression. Group information and DE value are included for each gene.

File name: Supplementary Data 7.
Description: Gene lists for female (f)- and carcass (c)-associated expression. Group information and DE value are included for each gene.

File name: Supplementary Data 8.
Description: Gene lists for male (m)- and reproductive (r)-associated expression. Group information and DE value are included for each gene.

File name: Supplementary Data 9
Description: Gene lists for male (m)- and carcass (c)-associated expression. Group information and DE value are included for each gene.

File name: Supplementary Data 10.
Description: Annotation of genes with the 'No change' and 'Other' expression patterns in reproductive organs and carcasses of intersexes compared with normal females. Top 20 highly expressed genes or all genes, if the total gene numbers are less than 20, were selected in each group.

File name: Supplementary Data 11.
Description: Annotation of sex and tissue- specific genes with 'No change', 'Other', and 'Down-regulation' expression patterns. (a-c) Female- specific/reproductive specific genes with 'No change', 'Other', and 'Down-regulation' expression patterns, respectively. (d-f) Female-specific/carcass specific genes with 'No change', 'Other', and 'Down-regulation' expression patterns, respectively. (g-i) Male-specific/reproductive specific genes with 'No change', 'Other', and 'Down-regulation' expression patterns, respectively. (j-l) Male-specific/carcass specific genes with 'No change', 'Other', and 'Down-regulation' expression patterns, respectively.

File name: Supplementary Data 12.
Description: Enriched GO terms of male-specific/reproductive-specific genes with different expression patterns. (a) GO enrichment analysis of all male-specific and reproductive-specific genes using all Ae. aegypti genes as a background. GO terms only with 'No change' expression pattern are in gray color. (b) GO enrichment analysis of genes with the 'Down-regulation' pattern using all male-specific and reproductive specific genes as a background.

File name: Supplementary Data 13.
Description: Tissue and stage associated gene expression data of genes in the group of male- and reproductive-specific genes with 'Down-regulation' expression pattern. MAG-enriched genes are highlighted in green color. The data was obtained from Vector Base (https://vectorbase.org/vectorbase/app).

File name: Supplementary Data 14.
Description: Sample IDs in RNAseq experiments. M, Ae. mascarensis; U, Ae. aegypti Uganda; UUM, backcrosses between female U and F1 males from crosses between female U and male M; f, female; m, male; ix, intersex males; C, carcass; R, reproductive organs.

File name: Supplementary Movie 1.
Description: Motile sperm, spermatheca, and mature ovarioles in the reproductive organ of an abnormal male (corresponds to Supplementary Figure 2b).
